# Quantum Dot Based Nano-Biosensors for Detection of Circulating Cell Free miRNAs in Lung Carcinogenesis: From Biology to Clinical Translation

**DOI:** 10.3389/fgene.2018.00616

**Published:** 2018-12-06

**Authors:** Radha D. Singh, Ruchita Shandilya, Arpit Bhargava, Rajat Kumar, Rajnarayan Tiwari, Koel Chaudhury, Rupesh K. Srivastava, Irina Y. Goryacheva, Pradyumna K. Mishra

**Affiliations:** ^1^Department of Molecular Biology, ICMR-National Institute for Research in Environmental Health, Bhopal, India; ^2^School of Medical Science and Technology, Indian Institute of Technology, Kharagpur, India; ^3^Department of Biotechnology, All India Institute of Medical Sciences, New Delhi, India; ^4^Department of General and Inorganic Chemistry, Saratov State University, Saratov, Russia

**Keywords:** lung cancer, circulating nucleic acids, environmental health, translational medicine, nanobiosensor, circulating miRNAs, quantum dots, translational research

## Abstract

Lung cancer is the most frequently occurring malignancy and the leading cause of cancer-related death for men in our country. The only recommended screening method is clinic based low-dose computed tomography (also called a low-dose CT scan, or LDCT). However, the effect of LDCT on overall mortality observed in lung cancer patients is not statistically significant. Over-diagnosis, excessive cost, risks associated with radiation exposure, false positive results and delay in the commencement of the treatment procedure questions the use of LDCT as a reliable technique for population-based screening. Therefore, identification of minimal-invasive biomarkers able to detect malignancies at an early stage might be useful to reduce the disease burden. Circulating nucleic acids are emerging as important source of information for several chronic pathologies including lung cancer. Of these, circulating cell free miRNAs are reported to be closely associated with the clinical outcome of lung cancer patients. Smaller size, sequence homology between species, low concentration and stability are some of the major challenges involved in characterization and specific detection of miRNAs. To circumvent these problems, synthesis of a quantum dot based nano-biosensor might assist in sensitive, specific and cost-effective detection of differentially regulated miRNAs. The wide excitation and narrow emission spectra of these nanoparticles result in excellent fluorescent quantum yields with a broader color spectrum which make them ideal bio-entities for fluorescence resonance energy transfer (FRET) based detection for sequential or simultaneous study of multiple targets. In addition, photo-resistance and higher stability of these nanoparticles allows extensive exposure and offer state-of-the art sensitivity for miRNA targeting. A major obstacle for integrating QDs into clinical application is the QD-associated toxicity. However, the use of non-toxic shells along with surface modification not only overcomes the toxicity issues, but also increases the ability of QDs to quickly detect circulating cell free miRNAs in a non-invasive mode. The present review illustrates the importance of circulating miRNAs in lung cancer diagnosis and highlights the translational prospects of developing QD-based nano-biosensor for rapid early disease detection.

## Introduction

Lung cancer, also known as bronchogenic carcinoma, is considered as a serious public health concern and a leading cause of mortality in Southeast Asia. The disease globally contributed to about 11.6% of all newly diagnosed cancer cases and 18.4% of the total mortality rate in 2018 (Bray et al., 2018). Importantly, the disease prevalence and mortality rates are higher in developing or under-developed regions of the world and positively correlate with the socio-economic status of the affected population (Wong et al., 2017). The disease is primarily categorized as SCLC which contributes to 13% of the total lung cancer cases and NSCLC that contributes to 85% of total reported cases. SCLC is localized to primary and secondary bronchi and is highly metastatic, whereas NSCLC often occurs in the lung peripheral tissues and is comparatively easy to detect (Collins et al., 2007). NSCLC is further categorized on the basis of histopathology and classified as squamous cell carcinoma, adenocarcinoma, and large cell carcinoma (Howlader et al., 2013). The higher mortality observed among lung cancer patients, is attributed to the delay in disease diagnosis. The overlapping symptoms with other respiratory conditions such as chronic obstructive pulmonary disease often lead to the disease being unidentified or misdiagnosed (Bjerager et al., 2006; Mackillop, 2007). On the other hand, the effect of suggested screening test, LDCT, on overall mortality has not been statistically significant (Aberle et al., 2011). In addition, the use of LDCT as a reliable community-based screening technique is limited by its excessive cost (Wildstein et al., 2011; Goulart et al., 2012) and exposure associated risk to harmful radiations (Brenner, 2012; McCunney and Li, 2014). Therefore, identification of specific biomarkers, capable of detecting the presence of malignancy at an early stage might help to reduce mortality.

## Current Serological Biomarker for Detection of Lung Cancer

Owing to the high metastatic potential of lung cancers a number of protein molecules have been assessed to identify the optimum serological marker for the early cancer detection (Pan et al., 2017). Although a majority of these markers have been found to work efficiently under regulated conditions, the clinical success of these markers is broadly restricted by the modulating behavior of tumor cells. Ideally, the establishment of a potent serological biomarker requires extensive molecular profiling of the tumor samples and subsequent validation. CEA is a cell surface glycoprotein which is broadly involved in different intracellular signaling and cell adhesion processes. Normally absent or under-expressed in adults, this oncofetal protein is significantly over expressed during malignant conditions and is shown to be a well-characterized tumor biomarker for lung cancer screening (Blankenburg et al., 2008; Tomita et al., 2010; Hanagiri et al., 2011). SAA, a family of inflammation associated apolipoproteins are reported to have an important function in driving cells to metastasis (Cho et al., 2010; Sung et al., 2011; Biaoxue et al., 2016). The analyzed sensitivity for SAA based ELISA ranges between 50 and 70% with 95% specificity (Sung et al., 2011, 2012). Similarly, a threefold escalation in the levels of Hp β, a free hemoglobin-binding glycoprotein was reported in lung cancer patients’ sera. As the Hp β chain encompasses significant stability, it may act as a prominent diagnostic indicator for lung cancer (Kang et al., 2011). The utility of complement C9 proteins and endoglin as favorable lung cancer biomarker has been also established (Narayanasamy et al., 2011; Kopczynska et al., 2012). Moreover, elevated levels of growth factor MDK (Xia et al., 2016; Jing et al., 2017), tissue inhibitor of metalloproteinase (TIMP1) (Jumper et al., 2004; An et al., 2015), IGFBP-2 (Zhang Y. et al., 2013) and serine protease TFPI (Fei et al., 2017), has been also evidenced in the systemic circulation of lung cancer patients. Likewise, the squamous cell carcinoma antigen, a structural cytoplasmic protein, tumor M2- pyruvate kinase (PKM2) (Schneider, 2006) and CYFRA 21-1 (Kulpa et al., 2002) are also detected to be elevated among lung cancer patients with a critical diagnostic potential. A complete list of proteins suggested to be used as probable serum biomarkers for lung cancer diagnosis is summarized in Table 1. Although these proteins may be useful, however, low abundance, high mutation rate, and complex interactions with other serum proteins limit their application in mass screening.

**Table 1:** Serological biomarkers for lung cancer detection.

Biomarker	Function	Reference
Carcinoembryonic antigen	Cell adhesion, regulation of signal transduction and innate immunity.	Hammarstrom, 1999; Kuespert et al., 2006; Tiernan et al., 2013
Serum Amyloid A	Synthesize several cytokines, bind and activate cell surface receptors and activate inflammasome cascade.	Urieli-Shoval et al., 2000; Eklund et al., 2012
Haptoglobin β chain	Immunomodulatory in nature which also shows hemoglobin-binding capacity and is α-sialoglycoprotein in an acute phase.	Kang et al., 2011; Vanuytsel et al., 2013
Anti-insulin-like growth factor-binding protein-2	Modulates insulin-like growth factor actions by preventing its binding to the receptor along with regulation of growth hormone-stimulated growth, cell proliferation and adhesion.	Polticelli et al., 2008; Shen et al., 2012
Complement component 9 proteins	Forms pores on the membrane of pathogens. Also plays a role in inducing tolerance, destruction of apoptotic cells and clearance of immune complexes.	Dudkina et al., 2016; Xiao et al., 2017
Endoglin	Regulation of ALK1-dependent adhesive and proliferative effects.	Lebrin et al., 2004; Koleva et al., 2006
Midkine	Growth factor which plays a role in promoting growth, gene expression, migration, survival, reproduction, and repair.	Maeda et al., 1999; Yoshida et al., 2001
Tissue Inhibitors of Metallo-proteinases	Bind to the Matrix metalloproteinases and inactivate them. They also play a role in degradation of Extracellular Matrix.	Lose et al., 2005; Louhelainen et al., 2010
Tissue Factor Pathway Inhibitor	Regulates the initiation phase of blood coagulation by hindering factor Xa and the tissue factor/factor VIIa complex. Matrix metalloproteinase, plasmin, cathepsin G, trypsin, and plasma kallikrein are some of the enzymatic activities negatively regulated by Tissue Factor Pathway Inhibitor.	Augustsson et al., 2014; Li et al., 2017
Squamous Cell Carcinoma Antigen	Represses the natural killer cell induced apoptosis, stimulates synthesis of matrix metalloproteinase-9 production whereas suppression of squamous cell carcinoma antigen induces cell cancer invasion and relocation in disease expression of *E*-cadherin.	Murakami et al., 2010; Catanzaro et al., 2011
Tumor M2- pyruvate kinase	Extremely active tetrameric protein which on exposure to oncoproteins, converts to less active dimeric form which is an important step for tumor metabolism.	Sithambaram et al., 2015; He et al., 2016
CYFRA 21-1	A cytokeratin-19 fragment which is used as a tumor marker.	Okamura et al., 2013
C-reactive protein	Produced during injury and employs anti-arthritic, anti-pneumococcal, anti-atherosclerotic, and anti-amyloidogenic functions as well.	Salzberg et al., 2006; Thirumalai et al., 2017
Human Plasma Kallikrein	Participates in inflammation and intrinsic blood coagulation system and also regulates vascular responses.	Liu W. et al., 2016; Ottaiano et al., 2017
Methylated DNA	Hallmark of cancer which occurs very early during cancer development.	Fujita et al., 2014; Widschwendter et al., 2017
Intercellular Adhesion Molecule 1	Promotes adhesion, transendothelial migration and immunological synapse formation.	de Paula et al., 2017; Marzolla et al., 2017
Pro gastrin releasing peptide	A specific tumor marker. Mitogenic activity of proGRP has also been demonstrated.	Shibayama et al., 2001; Oremek and Sapoutzis, 2003; Oh et al., 2016
Urokinase-type plasminogen activator receptor	Plays a vital role in plasminogen activation and fibrinolysis. Also involved in cardiovascular diseases.	Semina et al., 2016; Yousif et al., 2018
Progesterone Receptor Membrane Component 1	Essential for ovulation and viability of granulosa cells of preovulatory follicles. *In vitro* studies have shown its role in tumor proliferation.	Peluso et al., 2008; Kowalik et al., 2016; Zhao et al., 2017
Matrix metalloproteinase 1	Plays a major role in inflammatory processes and extracellular matrix breakdown and has been associated with tumor invasion and metastasis.	Sauter et al., 2008; Sousa et al., 2014
Epithelial cell adhesion molecule.	Functions as a cell-cell adhesion molecule in order to maintain cell polarity. Also involved in the proliferation and differentiation. Used as a marker for stem/progenitor cells.	Terris et al., 2010; Wen et al., 2018


*CYFRA 21-1, Cytokeratin 19 fragments; ALK1, Activin-like kinase receptor 1.*

## miRNA: Biogenesis and Classification

**FIGURE 1:**
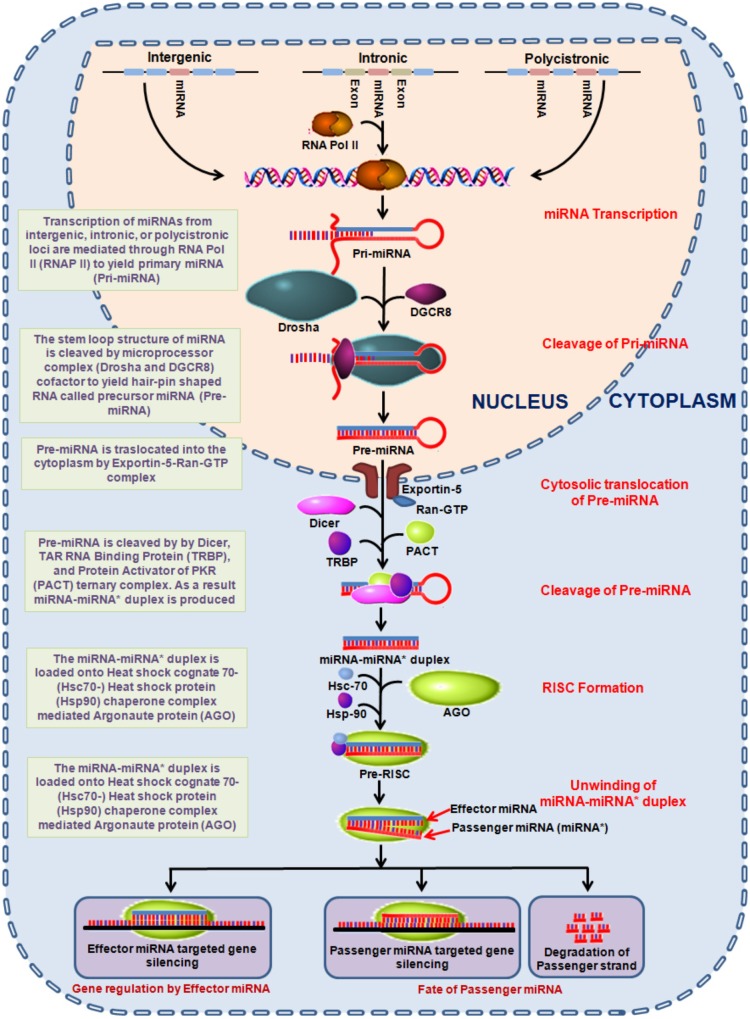
Diagrammatic representation of miRNA biogenesis. miRNA encoded genes transcribe to produce primary miRNAs which undergo a series of endolytic maturation steps to produce the mature effector miRNA involved in gene regulation. AGO, argonaute; Hsp, heat shock proteins; miRNA, mircoRNA; PACT, protein activator of the interferon-induced protein kinase; Ran-GTP, Ras-related nuclear protein-guanidine adenosine tri-phosphate; RISC, RNA-induced silencing complex; TRBP, TAR RNA binding protein.

Epigenetic alterations involving DNA methylation, histone modification and microRNAs (miRNAs) are significantly associated with vital signaling cascades and differentially reflect in various malignancies including lung cancer (Cheng, 2015; Larrea et al., 2016; Cruz et al., 2017; Ma et al., 2018). Out of which, miRNAs are considered vital to establish a clinically relevant minimal-invasive markers for risk assessment, early detection and monitoring therapeutic responses. miRNAs are small 19–22 nucleotide long non-coding RNAs which plays a crucial role in the regulation of gene expression. miRNAs are encoded within the nuclear genome and function via base pairing with the complementary mRNA molecule. As a result, the targeted mRNA is silenced either by cleaving, destabilizing (via polyA tail shortening) or manipulating the translational efficiency through ribosome mediated mRNA targeting. The miRNA encoded genes, usually found in sense orientation are transcribed by RNA polymerase II. The transcribed miRNA (∼80 nucleotides) forms a stem-loop structure, primarily known as pri-miRNA which is processed in the nucleus to generate precursor-miRNA (pre-miRNA). This is followed by a series of enzymatic reactions (Lee et al., 2003) and then translocation from the nucleus to cytoplasm (Lund et al., 2004) to further yield mature miRNA (Hutvagner et al., 2001) as shown in Figure 1. These mature miRNAs are then loaded onto the AGO to form an effector miRNA-induced silencing complex +(miRISC). The passenger strand is either degraded or remains loaded with RISC complex (Schwarz et al., 2003). The effector miRNA guides miRISC to target mRNA, where the degree of miRNA–mRNA complementarity determines the process of miRNA mediated gene silencing mechanism (Figure 1) (Ambros, 2004). Moreover, the ability to regulate gene expression of a number of genes by a single miRNA can comprehensively influence a wide array of fundamental cellular processes; for instance proliferation, growth, cell differentiation, mobility, and apoptosis (Ambros, 2004). This is imperative as miRNAs conceivably possess the oncogenic or tumor suppressing ability and may thus assume an indispensable part in both initiation and regulation of processes related to carcinogenesis (Esquela-Kerscher and Slack, 2006; Zhang et al., 2007).

In general, miRNAs are classified on the basis of their function, as tumor suppressors or oncogenes. The oncogenic miRNAs are known as “oncomirs” and primarily contributes toward tumor progression via negatively influencing the expression of genes regulating cell differentiation or apoptosis. While the other class, that play a major role in inhibiting the oncogenes are characterized as tumor suppressive miRNAs (Table 2) (Esquela-Kerscher and Slack, 2006; Zhang et al., 2007).

**Table 2:** Classification of miRNAs.

miRNA	Targets	Overview	Reference
**Tumor Suppressor miRNA**			
Let-7 Family	Members of Ras family (H-ras, K-ras, and N-ras) and high mobility group A (HMGA2)	(1) Play a crucial role in cell division and differentiation (2) Mature Let-7 family miRNAs are processed from 13 precursor sequences (3) The genes encoding these miRNAs are primarily located in the regions that are often deleted during (4) Let-7a and Let-7f are known to portray a foremost role in the functional regulation of oncogenes (5) Ectopic expression of let-7 miRNA is observed to repress cellular proliferation	Calin et al., 2004; Johnson et al., 2005; Sarhadi et al., 2006; Koscianska et al., 2007; Lee and Dutta, 2007; Roush and Slack, 2008; Shi et al., 2009; Boyerinas et al., 2010; Wong et al., 2011

miR-34 family	Suppresses p53 inhibitors and enhances p53 protein stability	(1) The miR-34 family is produced from two different transcriptional units which collectively comprises of three members, i.e., miR-34a, miR-34b and miR-34c (2) Gene encoding miR-34a is located on chromosome 1p36, while miR-34b and miR-34c are co-transcribed on chromosome 11q23 (3) Frequent deletions of 1p36 and translocation, insertion, and inversion of 11q23 region has been reported in lung cancer cases. (4) These rearrangements results in down-regulation of mir-34 and up-regulation of proto-oncogenes like *MYC* and *BCL2*, and causes deregulated cellular proliferation and apoptosis (5) miR-34 are vital regulators of EGFR signaling pathway and are often down-regulated in NSCLC	Dave et al., 1999; Nomoto et al., 2000; Bommer et al., 2007; Gazdar and Minna, 2008; Hermeking, 2010; Kaller et al., 2011; Kasinski and Slack, 2012; Mandke et al., 2012; Garofalo et al., 2013; Adams et al., 2016

miR-200 family	Transcriptional repressors of E-cadherin ZEB1 and ZEB2	(1) The miR-200 family situated on chromosomes 1 and 12 in the human encompasses five members organized as two clusters, miRs-200b/a/429 and miRs-200c/141 (2) The inter-cluster expression of these miRNAs correlates with each other while their intra-cluster expression does not appear to be highly correlated (3) The expression of miR-200 family have manifested marked downregulation in lung cancer cells that have undergone EMT	Gregory et al., 2008; Korpal et al., 2008; Park et al., 2008; Ceppi et al., 2010; Takeyama et al., 2010

**Oncogenic miRNA**			
miR-21	PTEN, RECK and Bcl-2	(1) miR-21 inhibits the activity of phosphatases thereby downregulating the negative regulators of RAS/MEK/ERK pathway to drive the process of tumorigenesis by stimulating cellular proliferation and the process of invasion, and metastasis	Jung and Calin, 2010; Zhang J.G. et al., 2010; Haffner et al., 2013; Yang Y. et al., 2015; Sims et al., 2017


miR-17-92 cluster	E2F1, BCL2L11, HIF1A, and PTEN	(1) The miR-17-92 cluster is likewise named as human oncomiR-1 and encompasses seven members (miR-17-5p, miR-17-3p, miR-20a, miR-18a, miR-92a, miR-19a, and miR-19b) (2) c-Myc is reported to directly target mir-17-92 cluster, and transcriptional regulation is significantly influenced by expression profile of Myc proto-oncogene (3) Notch and Sonic Hedgehog pathways are also involved in the activation of miR-17-92 in cancer (4) A higher miR-17-5p expression contrarily relates with the survival of lung cancer patients	Hayashita et al., 2005; Matsubara et al., 2007; Osada and Takahashi, 2011; Li et al., 2014

miR-221/222	PTEN, TIMP3, BIM, RB1, p21, and p27	(1) These miRNAs are reported to be associated with the aggressiveness of lung cancers (2) Its over-expression down-regulates PTEN and TIMP3 to inhibit apoptosis and promotes cell migration (3) Interaction of extracellular HMGB1 with RAGE results in enhanced miR-221/222 cluster expression which sequentially inhibits tumor suppressor gene PTEN to facilitate tumor escape	Garofalo et al., 2009; Mardente et al., 2015


## miRNAs in Circulation

**FIGURE 2:**
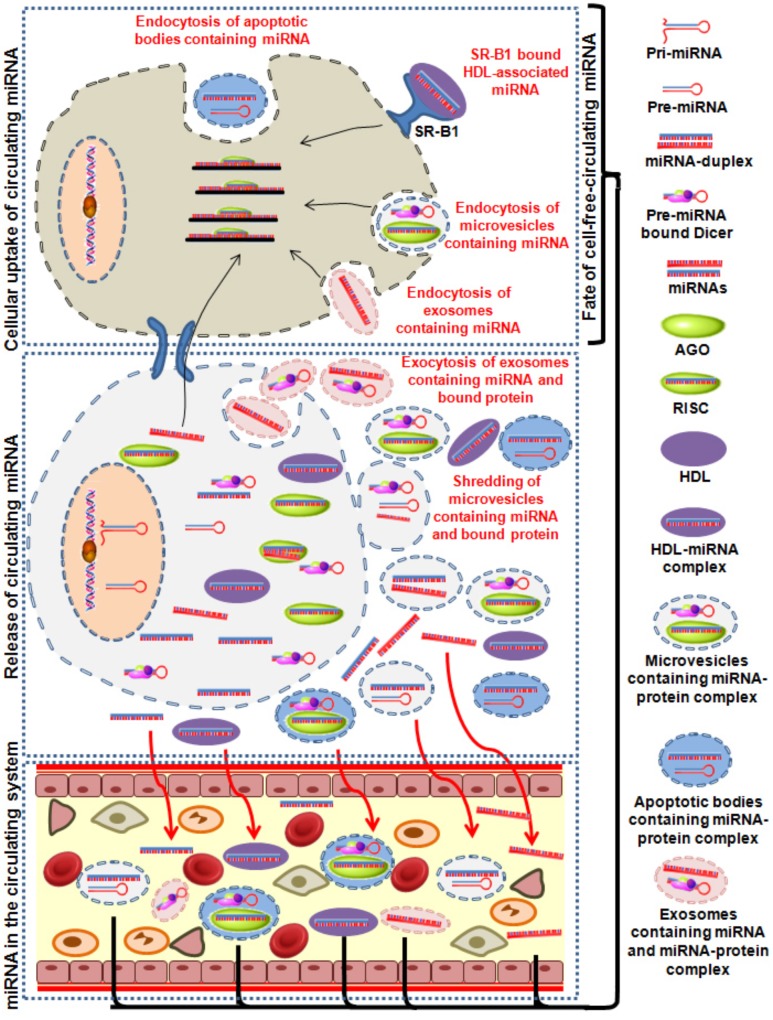
Image showing the summarized sequence of different processes which causes release of miRNAs in circulation. Release of miRNA containing exosomes, microvesicles and apoptotic bodies and protein bound miRNA into the excellular medium is either passively or actively taken up by the recipient cells. AGO, argonaute; HDL, high-density lipoproteins; miRNA, mircoRNA.

The vital role of miRNAs in many biological processes is evident by its ability to regulate gene expression. About 700 human miRNAs with each having up to hundreds of novel target mRNAs has been identified. In addition to their endogenous origin, miRNAs are also reported to be present in extracellular matrices (Figure 2). Following primary reporting of circulating mRNAs in human plasma, the presence of extracellular miRNAs were also reported in the bloodstream (Mitchell et al., 2008) ensued by occurrence of tumor-associated miRNAs in lymphoma patients (Lawrie et al., 2008; Fernandez-Mercado et al., 2015) and circulating placental miRNAs among pregnant women (Chim et al., 2008). Eventually, the existence of these circulating miRNAs was also reported in blood plasma (Mitchell et al., 2008), serum (Chen et al., 2008; Lawrie et al., 2008), saliva (Park et al., 2009), urine (Hanke et al., 2010), and other body fluids. However, depending on the health status and disease etiology the concentration of miRNAs may significantly vary (Weber et al., 2010). Liberation of extracellular miRNAs from cells might be associated with passive leakage due to tissue injury (Chen et al., 2008; Mitchell et al., 2008) or with RNA-binding protein (Wang et al., 2010; Arroyo et al., 2011; Vickers et al., 2011) or through dynamic release in the microvesicular framework (Valadi et al., 2007; Zernecke et al., 2009; Zhang Y. et al., 2010). These molecular entities supposedly originate from blood cells, circulating tumor cells (Heneghan et al., 2010; Zhao et al., 2010) or other disease affected tissue cells (Chen et al., 2008; Chin and Slack, 2008) and possess the ability to reflect the ongoing patho-physiological conditions. It has been demonstrated that discrete pathological conditions such as lung cancers (Chen et al., 2008; Lawrie et al., 2008; Mitchell et al., 2008; Park et al., 2009; Hanke et al., 2010), tissue injuries (Ji et al., 2009; Laterza et al., 2009; Wang et al., 2009) and diabetes (Chen et al., 2008) can be likewise altered due to difference in the levels of the circulating miRNA concentrations (Park et al., 2009; Hanke et al., 2010; Weber et al., 2010).

## Packaging and Release of Extracellular Circulating miRNAs

Despite resistance against RNase activity circulating miRNAs are highly susceptible to detergents (El-Hefnawy et al., 2004; Zhang Y. et al., 2010) or proteinase K activity (Turchinovich et al., 2011) indicating the presence of membrane envelop and other proteins. The presence of a membrane-bound miRNA and different mechanisms for packaging and transport of these extracellular miRNAs have also been reported. These mechanisms include miRNAs encapsulation in membrane-derived vesicles, RNA-binding proteins, or lipoprotein complexes such as HDL.

## Membrane-Derived Vesicles

Exosomes initially discovered by Pan and Johnstone, are small (50–90 nm) homologous secreted membrane vesicles of endosomal origin which serve as miRNA carter. The formation of these lipoprotein particles primarily involves the fusion of plasma membrane with multivesicular bodies and relies on the calcium influx, calpain and cytoskeleton reorganization (Yanez-Mo et al., 2015). The packaging of exosomal miRNAs requires enzymatic activation and is an active energy (ATP) dependent cellular mechanism which can function as intercellular miRNA transmitter between cells. Moreover, larger membrane vesicles also known as microvesicles are produced by direct budding from the plasma membrane and play an integral role in cell-to-cell miRNA transfer. These particles differ from exosomes on the basis of their release mechanism, biogenesis and biophysical properties. Microvesicle formation occurs through outward budding and fission of membrane vesicles which is contrasting to the formation of exosomes via inward budding. The particles directly shed from the plasma membrane and possess almost similar contents; however, the release is more heterogeneous in nature (i.e., particles of 100–1000 nm) (Aharon et al., 2009; Chironi et al., 2009; Lee et al., 2012). Apoptotic bodies which are liberated from apoptotic cells as a repercussion of programmed cell death can also contribute to the active secretion of miRNAs. These are the largest extracellular vesicles and their size may vary from 1 to 5 μm (Turiak et al., 2011). Similar to other extracellular vesicles, apoptotic bodies have demonstrated to carry various different molecules including miRNAs.

## RNA-Binding Proteins

Apart from the membrane-derived vesicles, circulating miRNAs are also observed to be associated with RNA-binding proteins. Initially, NMP1, a nucleophosphoprotein was shown to protect miRNA from RNase degradation but later circulating miRNAs were also shown to bind with AGO family (mainly AGO2) (Wang et al., 2010; Arroyo et al., 2011). These secreted circulating miRNAs are dynamically released from cells into the plasma in association with Argonaute2 (AGO2) without being encapsulated in microvesicles (Arroyo et al., 2011; Turchinovich et al., 2011). AGO2 is a major component of RNA-induced silencing complex (RISC) which binds with functionally mature miRNAs to regulate translation of cellular mRNAs. Studies have suggested that only 10% of the total cell-free miRNAs released in plasma is through microvesicles while approximately 90% of the circulating miRNAs is co-fractionated with ribonucleoprotein complexes (Arroyo et al., 2011).

## Lipoprotein Complexes

Circulating miRNAs in blood plasma are also reported to be attached to lipoproteins. These lipoprotein complexes are specifically rich in small non-coding RNAs and mainly involve LDL and HDL and plays a major role in the transportation of lipids and fat-soluble vitamins through the bloodstream (Vickers et al., 2011; Tabet et al., 2014). The higher affinity of lipoproteins to attach with water-insoluble materials enables these complexes to effectively carry nucleic acids in circulation (Kim et al., 2007; Babin and Gibbons, 2009). This also suggests a vital role of these lipoprotein complexes in the biogenesis of the lipoprotein itself. On the other hand, it has been insinuated that the relationship of miRNAs with HDL could be to some extent non-specific.

## Importance in Patho-Physiological Processes

The observations that circulating miRNAs can be conveyed from one cell to another evidenced their potential role in cellular communication (Valadi et al., 2007; Montecalvo et al., 2012; Xin et al., 2012). It is currently evident that variety of viable cells take up these circulating miRNAs regardless of their cellular origin. Studies have demonstrated that majority of such communication may involve the paracrine or autocrine mode of signaling. Initial reports showed that AGO2 protein associated ccf-miRNAs are broadly involved in such communication (Arroyo et al., 2011) but later studies have shown that the majority of ccf-miRNAs are in exosomal form (Gallo et al., 2012; Sohel et al., 2013; Noferesti et al., 2015). Evidence suggests a unidirectional miRNA transfer from T cells to recipient APCs via CD63^+^ exosomes during immune interactions (Mittelbrunn et al., 2011). Such transfer possesses the ability to fine-tune gene expression during cellular communications. The exosomal transfer of ccf-miRNA is reported to significantly down-regulate their target genes (Kogure et al., 2011; Montecalvo et al., 2012; Sohel et al., 2013). Importantly, exosomes from metastatic cancers were observed to be rich in let-7 family tumor-suppressive miRNAs, while no such enrichment was reported among the exosomes from other cells that suggested the exosomal mode of exporting tumor-suppressive miRNAs (Ohshima et al., 2010). *In vitro* studies have shown that the proliferation and angiogenic potency of the recipient cells was found to be elevated when exosomes from mesenchymal stem cells were administered in cardiac stem cells (Zhang et al., 2016). Similarly, relocation of exosome and microvesicles from human bronchial epithelial cells encourage myofibroblast differentiation in lung fibroblasts (Fujita et al., 2015). It has also been also demonstrated that the transfer of stromal cell exosomes to breast cancer cells can alter their radiation sensitivity (Boelens et al., 2014). Exosomal miRNA mediated transfer of adriamycin-resistant capacity to breast cancer cells has also been reported (Mao et al., 2016). Several reports have demonstrated the importance of communication between cancer cells and the surroundings through microvesicles (Muralidharan-Chari et al., 2010). Transfer of microvesicle mediated extracellular tumor cell miRNAs were shown to promote tumor growth among the normal recipient cells (Skog et al., 2008). Unconventionally, these miRNAs were also reported to possess the ability to activate RNA-sensing Toll-like receptors (TLR). A subsequent activation of TLR7 and TLR8 was seen when tumor-derived exosomes (miR-21 and miR-29a) were transferred to immune cells that resulted in activation of a pro-metastatic inflammatory response leading to tumor growth and metastasis (Fabbri et al., 2012).

## Circulating miRNA: a Potential Biomarker for Early Detection of Lung Cancer

The evidences defining the presence of disease-specific miRNAs in circulation during different pathophysiological conditions including lung cancers holds considerable promise to develop these circulating entities as a potent disease biomarker. Higher levels of circulating exosomal miRNAs signifying the various tumors stages were reported in patients with lung adenocarcinoma (Rabinowits et al., 2009). In addition, a set of 63 disease specific circulating miRNA were observed in the serum of 11 lung cancer patients (Chen et al., 2008). It has been shown that the circulating miRNA signatures in plasma possess the ability to discriminate between known lung cancers from healthy controls with high diagnostic accuracy. In comparison to controls, sequence analysis suggested almost 28 lost miRNAs and 63 new miRNAs in the serum of NSCLC patients. It was also reported that higher levels of two tumor-associated miRNAs (miR-25 and miR-223) may serve as the marker for NSCLC (Chen et al., 2008).

Further, disease-specific up-regulation in the levels of two miRNAs, i.e., miR-1254 and miR-574-5p was seen in patients with early stage NSCLC (Foss et al., 2011). Tang et al., observed a set of three aberrantly expressed miRNAs (miR-155, miR-21, and miR-145) in the plasma of lung cancer patients (Tang et al., 2013). Diagnostic efficacy in terms of sensitivity and specificity of specified miRNA panels-based arrays, i.e., 3-miR assay miR-205, miR-210 and miR-708 in sputum samples of lung cancer was observed to be 73 and 96% respectively which further improved with 4-miR assay (miR-486, miR-21, miR-200b, and miR-375), i.e., 81 and 93% respectively (Xing et al., 2010). In order to further improve the diagnostic efficacy, a six circulating miRNAs panel was assessed and identified to possess the ability to distinguish early stage NSCLC patients from healthy controls or chronic obstructive pulmonary disease (COPD) patients (Halvorsen et al., 2016). Similarly, higher levels of miR-29c was reported in the NSCLC patients, while the expression profile of seven serum miRNAs (let-7a, miR-146b, miR-155, miR-221, miR-17-5p, miR-27a, and miR-106a) were observed to be significantly down-regulated (Heegaard et al., 2012). Interestingly, levels of significantly altered 10 miRNAs panel (miR-20a, miR-24, miR-25, miR-145, miR-152, miR-199a-5p, miR-221, miR-222, miR-223, and miR-320) could identify NSCLC almost 33 months prior to its clinical diagnosis (Chen et al., 2012). In addition to the disease identification, circulating miRNAs levels were also reported to correlate with the patient survival. The levels of miR-125b significantly correlated with poorer prognosis of NSCLC patients and response to cisplatin-based chemotherapy (Yuxia et al., 2012; Cui et al., 2013). Likewise, the higher levels of circulating miR-22 also correlated with the poor therapeutic response to pemetrexed-based treatment in NSCLC patients (Franchina et al., 2014). The downregulated let-7i-3p and miR-154-5p miRNAs were strongly associated with smoking and related lung cancer (Huang et al., 2014). Collectively, these studies have suggested the potential ability of circulating miRNAs to be utilized as biomarkers for lung cancer detection. However, specific detection of these miRNAs may require development of sensitive strategies capable of identifying specific and desired miRNAs in circulation.

## Determination of Circulating Cell Free miRNA

Several methods have been employed for the detection of circulating miRNAs. The qPCR method has been routinely used for the detection of miRNA in different biological samples where miRNA template is amplified and measured using different fluorescent detection probes like SYBR Green and Taqman fluorescent chemistry. SYBR Green-based qPCR technique utilizes the intercalating ability of SYBR Green and eliminates the need for a separate probe for each miRNA of interest; however, it holds less specificity. While, Taqman chemistry probes are specifically designed for the target miRNA and are sensitive, but comparatively less cost-effective. Alternatively, the use of stem-loop RT primers and locked nucleic acid (LNA) assay for the detection of miRNAs through qPCR increases specificity and sensitivity. The qPCR reaction using stem-loop primers is specifically used for the detection of mature miRNAs. In addition, LNA assay based miRNA quantification also discriminates between the precursor and mature miRNA. This technique overall is very expensive as it requires high-end instruments. Furthermore, microarray-based platforms can be also used to identify the change in differential miRNA expression level in the circulating body fluids, in a single run and on the global scale. Various microarray platforms such as GeneChip (Affymetrix), miRCURY LNA (Exiqon), and SurePrint (Agilent) have been adapted for miRNA quantification. The platforms have outlined probes for specific miRNA. The major drawback of microarray technology is excessive cost and compromised reproducibility (Draghici et al., 2006; Sato et al., 2009). There is no single pervasive method for microarray information analysis. Moreover, data normalization of the microarray is time-consuming and difficult especially for miRNA due to its low abundance and weak expression (Wang and Xi, 2013; Wu et al., 2013).

Another broadly utilized approach to determine the whole genome pattern of miRNA expression is next generation sequencing (NGS) (Margulies et al., 2005; Eminaga et al., 2013). Several studies have been performed to detect the circulating miRNA in the lung cancer patients through NGS (Ma et al., 2014; Gallach et al., 2017). The common work process of NGS includes RNA isolation, library preparation, sequencing, and data analysis. NGS is notably a preferable approach for the identification of novel miRNA biomarkers, however, implementation of such technology in the clinical processes possesses serious limitations as it requires specialized equipment and reagents which are very costly. Moreover, the data analysis of NGS is very complicated and necessitates standardization and further validation through qPCR method and thus restricting its use in the clinical field.

One more technique is isothermal amplification process which is performed at a consistent temperature and exists in various structures including exponential amplification reactions, loop-mediated amplification, rolling circle amplification, duplex-specific nuclease signal amplification, and hybridization chain reaction (Zhao et al., 2015). This method is highly efficient but involves a complicated primer designing process. It likewise brings about linear amplification rather than exponential amplification, which isn’t perfect for the discernment of feebly expressed miRNAs (Dirks and Pierce, 2004).

Detection of miRNA through NIR imaging in the biological system preferably eliminates the need for PCR and other complicated techniques and its variants. In addition to this, it has several advantages over its optical imaging counterparts such as autofluorescence and photobleaching (Gong et al., 2016). The NIR-based detection method of miRNA was accomplished through QDs and nanoclusters. The circulating or cellular miRNAs depending on our domain of the study may be detected through QD-based nanobiosensors. Current studies have elucidated the early detection of isolated miRNAs associated with various pathological conditions including cancer by means of QDs. QDs have a wide excitation, narrow emission spectra, high quantum yield, and exceptional photochemical stability. This approach has been described for immunohistochemistry (Stroh et al., 2005), immunoassays (Beloglazova et al., 2014, 2016) and detection of proteins in the biological samples (Qiu et al., 2017; Shu and Tang, 2017; Zhang et al., 2017). These nanoparticles contribute high specificity, substantial surface area and stability to the miRNA nano-biosensors.

## QDs Based Nano-Biosensor for Cancer Detection

**FIGURE 3:**
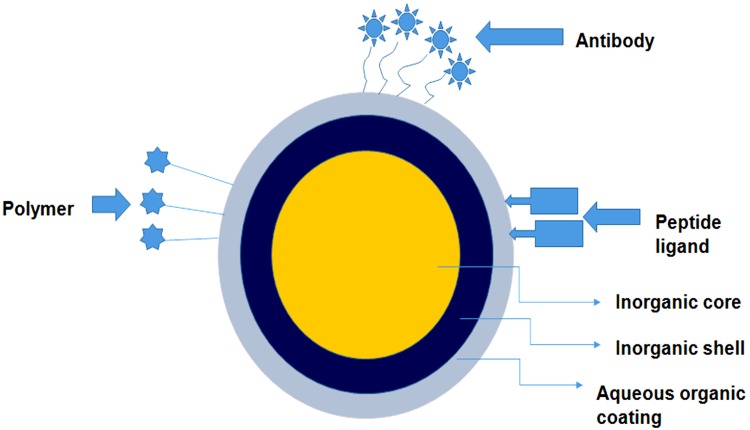
An overview of the in-depth structure of the quantum dot based nano-constructs. The figure illustrates that these nano-structures comprise an inorganic core, inorganic shell and an additional aqueous layer of polymers. The outer layer is layered with polymer or attached to desired peptide ligand/antibody for specific targeting.

Nanotechnology has been expanding its foothold in clinical medicine, especially in the area of oncology with a primary goal of early prognosis and diagnosis of the disease as well as real time monitoring of the progression in treatment. Nanomaterials exhibit excellent functional properties including huge surface-to-volume proportion for exceptionally proficient target interactions. These properties can be exploited to upgrade the execution of conventional techniques or to develop new measures with ultra-sensitivity and multi-parametric capacities. Nano-scale devices, particularly for the investigation of cancers involves the recognition of blood borne biomarkers such as cancer-associated proteins circulating tumor cells, circulating tumor DNA, and tumor- discarded exosomes. With the invention and advancements of nano-scaled sensorics, high sensitivity, specificity, multiplexing of measurements can be attained along with analysis at the genetic level for complete elucidation of patient’s stage and type of cancer as well as predicting proper treatment. Nanoparticles, such as superparamagnetic iron oxide and QDs are emerging as promising tools for medical imaging platforms. QDs are semiconductor nanosized crystalline structures (ranging from 2 to 10 nm) which possess superior fluorescent properties with less photobleaching (Figure 3). The size-tunable fluorescence of these nanocrystals spans all the way from QDs with a large diameter showing red fluorescence within the visible light spectrum to smaller diameter QDs with blue fluorescence. For this reason, QDs hold a broad excitation range coupled with a narrow and symmetric emission spectrum which enables QDs to get excited with a single wavelength and function in a multicolor mode for disease imaging and mapping (Tan et al., 2011). On the basis of their chemical composition, QDs can be subdivided into two broad categories. The first category is made up of elements from group III (Boron, Aluminum, Gallium) to V (Nitrogen, Phosphorous, Arsenic) of the periodic table, while the second category includes elements derived from subgroup II (Zinc, Cadmium) and the main group VI (Oxygen, Sulfur, Selenium) of the periodic group. The optimized quantum yield of the second category makes them superior and is thus preferred over first category QDs (Ghaderi et al., 2010).

## Synthesis of QDs

**FIGURE 4:**
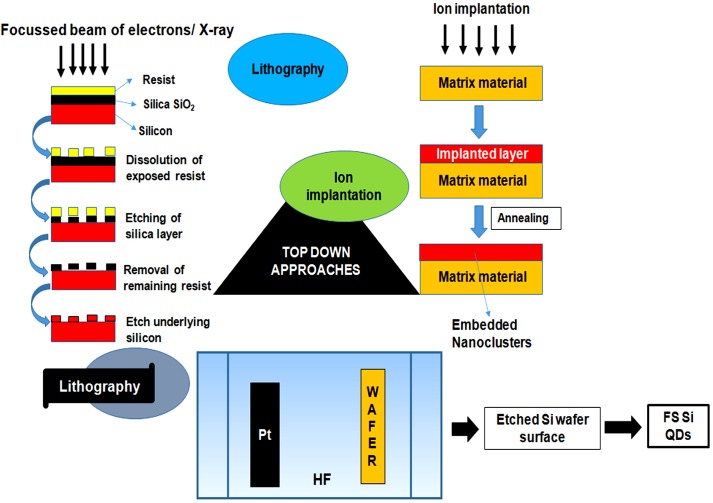
Figure showing the outline of top–down approaches for quantum dots synthesis. The approaches basically involve the step-by-step breakdown of a bulk piece of material to form particles in the nanometer range. Top–down approaches involves techniques like lithography, focussed ion beam techniques and etching techniques.

**FIGURE 5:**
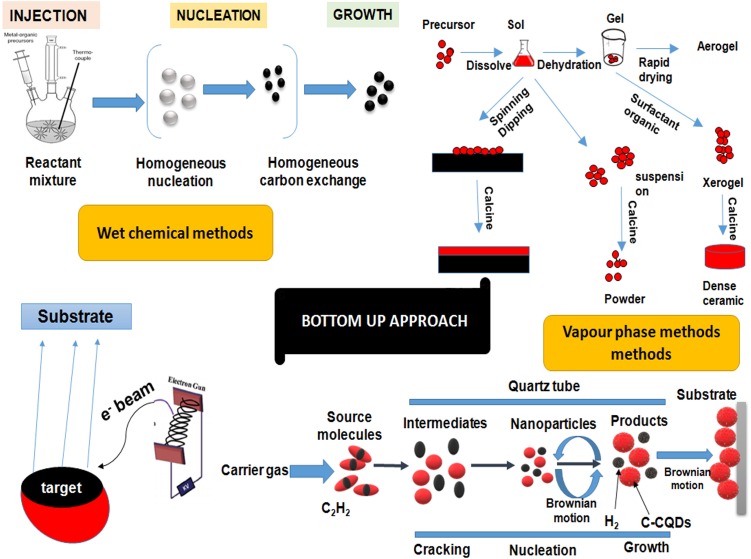
A diagrammatic representation of different bottom–up approaches for quantum dots synthesis. Bottom–up approaches involves the rearrangement and assembling of small atoms and molecules to achieve large nanostructures. Bottom–up approaches involves techniques like organometallic synthesis, sol-gel technique, sputtering, physical vapor deposition and chemical vapor deposition, etc. QDs, quantum dots.

Quantum dots are generally synthesized by using two approaches, i.e., Top–down approaches involving the breakdown of large initial structures through external forces and bottom–up approach involving the self-assemblage of miniaturized materials components via chemical reactions. Top–down processing comprehensively incorporates systems like lithography (e-beam and X-ray), MBE and ion implantation. QDs can be synthesized through different self-assembly (bottom–up) techniques, which might be additionally subdivided into wet-chemical and vapor-phase methods (Figures 4, 5). Wet-chemical methods are generally include techniques such as microemulsion, sol-gel, competitive reaction chemistry, hot-solution decomposition, sonic waves or microwaves, and electrochemistry, while in vapor-phase methods, layers are grown in an atom-by-atom process as a consequence of self-assembly that occurs on the substrate without any patterning (Valizadeh et al., 2012; Karmakar, 2015).

## Top–Down Processing Methods

The top–down approaches, lithography and FIB techniques are based on the principle of site specific etching or sputtering through bombardment of finely focussed, high energy beam of ions, photons, electrons or collimated (parallel) X-rays that causes dissolution and selective removal of exposed or covered regions of the resist material for the generation of fluorescent QDs (Wang et al., 2017; Weng et al., 2017). Several properties of QDs such as structure and inter-particle distance, primarily rely on the size of the ion beam (Mishra and Bhat, 2018). Another method known as etching, involves plasma formation through an interaction between gaseous molecules with a controlled radiofrequency current that results in break-down of the complex gas molecules to more reactive fragments. These high kinetic energy species further strike the surface and form a volatile reaction product to etch the patterned sample (Luo et al., 2014).

## Bottom–Up Approaches

### Wet-Chemical Methods

It includes traditional precipitation methods with cautious control of various parameters including mechanisms of nucleation, that may be homogeneous or heterogeneous wherein the antecedent particles amalgamate to a point till desired size is achieved and grow from a single solution or mixture. A general dialog of various wet chemical procedures is given beneath. These techniques include sol-gel process wherein a nanoparticle dispersed in a solution condenses to form a sol followed by polymerization to produce an elasticized gel. This further densifies and nucleates upon thermal treatment resulting in formation of particles with enhanced mechanical properties and structural stability. ZnO QDs prepared by using the sol-gel process have been reported (Bera et al., 2008; Yang P. et al., 2015). Another approach namely, microemulsion process involves the generation of nanometer water droplets using surfactants as a result of continuous exchange of reactants due to dynamic collisions. This acts as the limiting factor for growth of QDs. CdS QDs and two core-shell CdSe–CdS and CdSe–ZnS QD systems synthesized by reverse micelle technique was reported by Saran et al. (2012) and Damarla et al. (2016) respectively. The hot-solution decomposition method has been extensively used for the synthesis of QDs. This involves pyrolysis of a mixture of precursor and an organometallic compound such as tri-octyl-phosphine oxide (TOPO) at high temperature (∼300°C) under vacuum in a three-necked round bottom flask with vigorous stirring. This stimulates homogeneous nucleation to form QDs (21).

### Vapor-Phase Methods

In the vapor-phase method, layers of QDs are developed in an atom-by-atom process by the hetero-epitaxial growth of highly strained materials without any patterning. One such approach is MBE which involves heating of ultra-pure components like gallium and arsenic in semi Knudsen emanation cells until evaporation. These particles get consolidated on the wafer, where they respond with each other to frame single crystal gallium arsenide (Karmakar, 2015). Another technique comprises of vapor deposition techniques by physical or chemical means. The physical vapor deposition includes bombardment with a beam of vaporized atoms or molecules of the precursor material from a solid or liquid source which is then conveyed in the form of plasma to the substrate where condensation takes place. The method primarily involves three most important techniques, i.e., sputtering, pulsed laser deposition (PLD) and thermal evaporation. Effective preparation of crystalline QDs of Nb_2_O_5_ (1–20 nm) has been reported using the PVD (Dhawan et al., 2014; Karmakar, 2015). Similar to PVD, CVD method involves chemical reactions that bring about a transformation of the precursor atoms/molecules in the gaseous phase that deposit as a solid film or powder on the substrate. CVD strategies might be subdivided into vapor phase epitaxy (VPE) where CVD is utilized to deposit single crystal film, MOCVD in which precursors are metal-natural species, PECVD where a plasma is utilized to intensify the reaction and low-pressure CVD (LPCVD) wherein the decay is done at low pressure (Karmakar, 2015).

## Surface Chemistry and Energy Transfer Mechanisms

**FIGURE 6:**
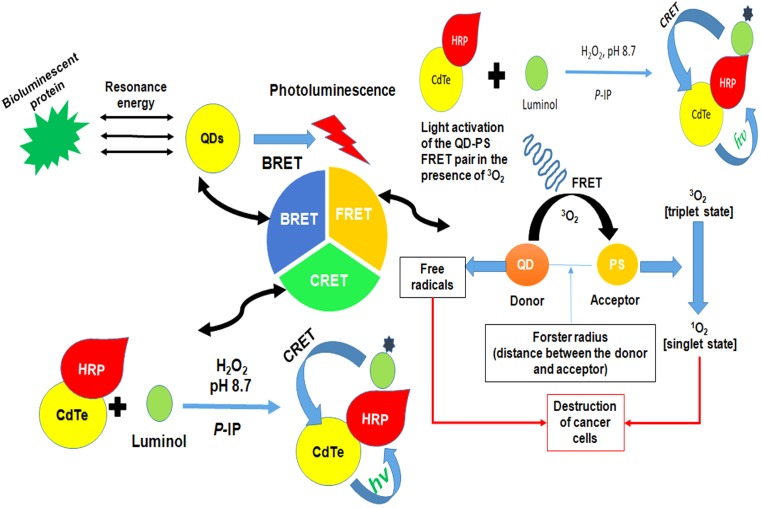
Figure demonstrating the general idea of different energy transfer schemes of quantum dots. FRET involves an energy transfer between two light sensitive molecules and the energy created by fluorescence excitation of one molecule is transferred to an adjacent molecule while, CRET involves transfer of resonance energy between chemiluminescence donor and quantum dot acceptors. In BRET energy transfer occurs between quantum dots and the luminescence donor. BRET, Bioluminescence resonance energy transfer; CRET, Chemiluminescence resonance energy transfer; FRET, Förster resonance energy transfer; HRP, horse radish peroxidase; QDs, quantum dots.

**FIGURE 7:**
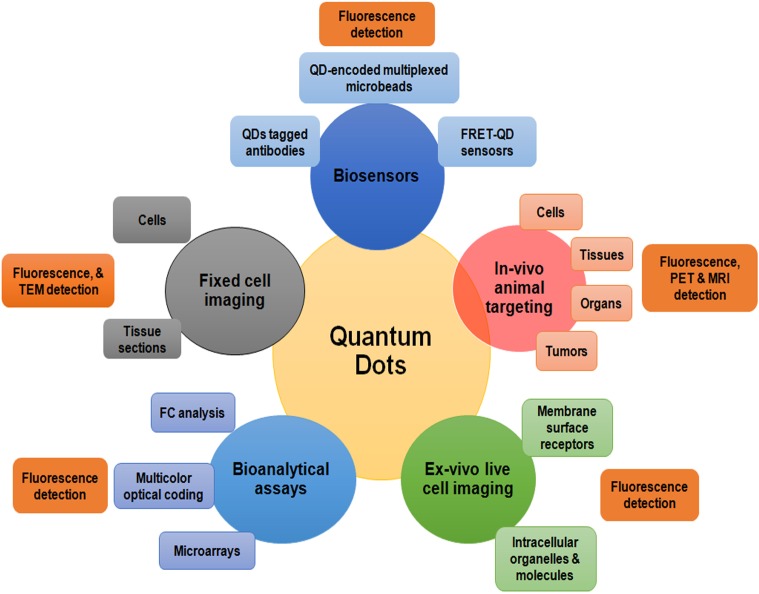
A summarized illustration of different quantum dots applications in the field of biomedical sciences. FRET; Förster resonance energy transfer; MRI, Magnetic resonance imaging; PET, Positron emission tomography; TEM, Transmission electron microscopy; QDs, quantum dots.

The surface modification by means of various bifunctional ligands or caps could improve the aqueous solubility, bio-compatibility and stability of QDs (whether single or core/shell structures). In core/shell QDs, the shell surrounding the core allows better passivation of surface defects and enhanced photostability with improved optical properties for instance in CdSe- ZnS QDs (Qu and Peng, 2002). Moreover, alloyed QDs allow continuous tuning of quantum confinement by variation in the QD size or their chemical composition. A number of methods have been employed for surface modifications of QDs such as introduction of a silica shell covering, exchange of the hydrophobic surfactant molecules with bifunctional molecules, or by coating with a cross-linked amphiphilic polymer. Other surface alteration techniques that have been widely assessed include electrostatic interaction, micelle encapsulation, and hydroxylation (Kakkar et al., 2013). QDs possess three classes of photon-induced energy transfer mechanisms which have been widely used for developing different QDs based strategies (Figures 6, 7 and Table 3).

**Table 3:** Energy transfer mechanism based Quantum Dots.

Quantum dots	Application	Mechanism	Reference
CdTe QDs	Detection of the breast cancer biomarker microRNA.	FRET	Borghei et al., 2017
Carbon QDs	Fluorescent platform for miRNA detection.	FRET	Khakbaz and Mahani, 2017
Graphene QDs conjugated with antibody anti-cardiac Troponin I	Detection of cardiac marker antigen Troponin I in blood based on FRET between conjugate and grapheme (quencher) only in 10 min.	FRET	Bhatnagar et al., 2016
Graphene QDs	Detection of miRNAs based on graphene quantum dots and pyrene-functionalized molecular beacon probes	FRET	Zhang et al., 2015
CdTe/CdS core-shell QDs capped with 3-mercaptopropionic acid	Ultrahigh-sensitive and -selective DNA and miRNA detection.	FRET	Su et al., 2014
Streptavidin-conjugated CdTe QDs	Exploration of the potential of far-red CdTe/ZnS core-shell Qdot705 (Invitrogen) as an efficient donor in a FRET assay.	FRET	Chong et al., 2007
CdTe QDs	An efficient CRET between luminol and QDs based on HRP–QD conjugates and the immuno-interaction of the QD–BSA and anti BSA–HRP in the luminol/hydrogen peroxide CL reaction.	CRET	Huang et al., 2006
CdSe/ZnSQDs.	Detection of ATP and DNA by the appropriate modification of QDs with nucleic acids capable of assembling the hemin/G-quadruplex DNAzyme upon detecting different analytes.	CRET	Freeman et al., 2011
CdSeTe/CdS QDs	NIR optical detection of apoptotic cells	BRET	Tsuboi and Jin, 2017
QD655	Bioluminescent QD was employed as an internal light source for meta-tetra-hydroxyphenyl-chlorin mediated photodynamic therapy	BRET	Hsu et al., 2012
CdSe/ZnS core-shell QD705	For lymph node mapping.	BRET	Kamkaew et al., 2016


*ATP, Adenosine-tri-phosphate; BRET, Bioluminescence resonance energy transfer; BSA, Bovine serum albumin; CdTe, Cadmium telluride; CRET, Chemiluminescence resonance energy transfer; FRET, Förster resonance energy transfer; HRP, Horse radish peroxidase; QDs, quantum dots; ZnS, Zinc sulfide.*

## Förster Resonance Energy Transfer

Förster resonance energy transfer, a technique widely used in assays and bioprobes, includes an energy transfer between two light-sensitive molecules known as FRET pairs. The energy exchange between FRET pairs takes place because of the long-range dipole-dipole interaction between them. QDs upon absorption of photons allow the transfer of their excitonic energy of electrostatically attached acceptor chromophores in close proximity in a non-radiative fashion through the long-range dipole-dipole coupling. The subsequent module outlines the utilization of FRET as an analytical signal. The obtained change in fluorescence can be used for attached moieties and thus, can be used as probes. Dissimilar to natural fluorophores, QDs display narrow emission and expansive absorption and do not permit the overlapping of absorption spectra of donor and acceptors, making them supreme and fantastic candidates for FRET applications. A basic requirement for the FRET is that the donor molecules emission spectrum must overlap absorption or emission spectrum of the acceptor. Moreover, they should possess almost parallel transition dipole presentations and the time of fluorescence generated by the donor molecule must be of adequate enough to let the FRET to occur. In addition, these molecules must be in close proximity, i.e., their distance must be less than 10 nm as the efficiency of energy transfer depends on the distance and the relation can be mathematically expressed by the equation: *E* = Ro 6/(Ro 6 + r6) where E is efficiency of energy transfer, Ro is Förster distance (the distance at which energy efficiency is 50%) and r is the donor-acceptor distance (Stanisavljevic et al., 2015). Thus, an ideal probe with efficient energy transfer depending upon the inter- or intra-molecular distances can be obtained by attachment of fluorophores to known sites within the molecules. This distance-dependence efficiency of FRET has been significantly evaluated for the assessment of structural dynamics, intermolecular association, biochemical events and disease diagnostics. One persistent advantage of the FRET chemistry is that both the donor and acceptor can be conjugated to antibodies (Pfleger and Eidne, 2006). This chemistry can be used to measure cell surface interactions while other signals cannot localize cellular fractionation. For instance, BRET cannot be used for single cell assessment as the signal produced is for entire cell rather than distinct cellular organelles. In addition, the strategy is relatively cheap and has no or fewer issues of bio-distribution and metal toxicity (Chandan et al., 2018).

## Chemiluminescence Resonance Energy Transfer

QDs due to their broad excitation spectra, large Stoke’s shifts, and size-dependent emission are well-matched fluorescent acceptors in CRET processes. The variable emission wavelength of QD-acceptors enables multiplex analysis, as they can be excited by the same chemiluminescent (CL) donors. QDs based-CRET occurs by the immediate oxidation of a luminescent donor when chemically produced excitons experience relaxation with emanating radiant energy. Due to the redox property of the discrete electron and hole states of QDs, they may act as catalysts in reactions involving other fluorophores (luminescent molecules). Amid the generation of hole into the valence band and electron into the conduction band by means of strong oxidizer and reducer respectively, short-lived radical species are produced that initiates oxidation of the CL reagents producing an enhanced CL. In addition, QDs can act as emitter species after CRET. Direct oxidation occurs when QDs are the only luminescent compounds in a CL system. At the point where more than one luminophore exists in a CL framework, the process can be either CRET (final emitter is a QD) or a catalytic process (final emitter is a luminophore) (Chandan et al., 2018).

## Bioluminescence Resonance Energy Transfer

Bioluminescence resonance energy transfer is a naturally prevailing event that includes non-radiative exchange of energy from a light discharging protein to an acceptor atom in proximity. Wide absorption spectra of QDs enable them to be energized by nearly every single bioluminescent protein and the unique properties of QDs (i.e., broad excitation and size tunable emission) enable them to create many QD-BRET pairs. Through pairing of Luc8, a bioluminescent protein with QD605, QD655, QD705, and QD800, a multiplexed BRET imaging was achieved (Chandan et al., 2018).

## QDs for Lung Cancer Diagnosis and Therapeutics

The optimal properties, ability to resist photo-bleaching and real-time detection makes QDs an ideal candidate for biosensing applications. The functionalized QDs that initially incorporated the potential affinity of streptavidin to avidin with a complementary binding probe have been assessed for development of different biosensing strategies. For instance, the biosensors for DNA comprise of QDs possessing Cy5-labeled complementary reporter probe along with a biotinylated capture probe with target DNA (Banerjee et al., 2015). Further appropriate modifications have been done to improve the cancer diagnostic ability of these nano-biosensors. Despite of the several methods available for early lung cancer screening, diagnosis at the early stage can be difficult and prone to error. A number of approaches have been proposed for different types of cancers, a few of them assessed in lung cancer settings are discussed here. As it is widely known that the molecular imaging of cancer cells is vital for efficient tumor diagnosis and personalized medicine therefore, Zhang C. et al. (2013) suggested the use of DNA functionalized QDs as efficient fluorescence nanomaterial for lung cancer bioimaging. The use of anti-HER2 conjugated QDs was applied for immune-labeling of breast and lung cancer cells and was reported to be superior in a panel of lung cancer cells with the differential HER2 expression proposing their potential pertinence in early identification of cancer biomarkers (Rakovich et al., 2014). A comparative approach utilizing affibody secured QDs was revealed to be a promising method for the production of fluorescent nanoprobes for imaging of tumor targets (Zhao et al., 2016). Furthermore, designing probes to detect disease specific mutations is an attractive approach toward early clinical identification of the different cancers. QDs linked to EGFR mutation-specific antibodies were assessed and reported to be highly effective and sensitive as compared with traditional methods in disease diagnosis and therapeutic decision making for NSCLC patients (Qu et al., 2014). In an attempt to further improve the diagnostic ability of these molecules, a highly specific QDs based recognition-before-labeling strategy using DNA aptamers was developed. The aptamers first recognize target cells, followed by the addition of fluorescent QDs which bind with aptamers and identify the target cells. This is a simple approach which omits the need of complicated QD functionalization, and the possibility of steric hindrance (Wu et al., 2015).

Epigenetic signatures such as methylated DNA and miRNAs possess the ability to be utilized as effective biomarkers for the diagnosis of lung cancers. Zeng et al. (2014) showed the efficiency of a QD-based miRNA nanosensor to detect point mutation in mir-196a2, abnormally expressed in the lung tissues of NSCLC patients. Tang et al. (2015) proposed the use of single QD-based biosensor for DNA point mutation assay which comprises Cy5-labeled biotinylated probes that utilized FRET chemistry for early clinical diagnosis. Another simplistic QD-based FRET method for the identification of DNA methylation at first depends on methylation-sensitive enzymatic absorption of genomic DNA emanate by its amplification and FRET-based discernment. The strategy productively evaluated the methylation levels of three tumor silencer genes PCDHGB6, RASSF1A and HOXA9 and was proposed to be a potential technique for non-invasive early clinical diagnosis of cancers (Ma et al., 2015, 2016). Similarly, for early identification of NSCLC, Fan et al. (2016) demonstrated the utilization of nano-QD microarray for the recognition of serum miRNAs (miR-17b-5p, miR-19-3p, miR-15b-5p, miR-16-5p, miR-20a-5p, and miR-92-3p) as diagnostic biomarkers. In a novel attempt to develop a combinatorial approach for treating lung cancer cells, a QD based system was developed to simultaneously deliver siRNA along with the known anticancer drugs (carboplatin, paclitaxel, and doxorubicin). These altered QDs also accommodated different anticancer drugs via HP-CD modifications along with binding and transporting the siRNA through electrostatic interactions with l-Arg residues. These multifunctional QD nanocarriers were accounted to be more effective and hold substantial pledge to act as robust tools for combined therapy of lung cancer (Li et al., 2016). In addition, the studies have been also performed to develop multiplex immunoassay systems. Liu L. et al. (2016) used QD as biomarker labels for diagnosis of lung cancer by using bead-based microarray. A sandwich structure is formed between the magnetic beads and the QD probes through the specific interactions of antigen and antibody of the target proteins and was easy to operate and cost effective (Liu L. et al., 2016). Multicolor QDs were used as detection elements and micro-magnetic beads as immune carriers for developing a highly sensitive and discrete multiplexed fluoro-immunoassay of three lung cancer biomarkers CEA, cytokeratin 19 (CYRFA 21-1), and NSE (Wu et al., 2016). In a recent attempt to develop QD based nanosensors for lung cancer diagnosis, a novel QD-lateral flow test strips system was formulated which has the capability to simultaneously detect CYFRA 21-1 and CEA in human serum. This QD based detection system is a quick and highly specific sandwich immunoassay with an ability to detect antigens within 15 min and is expected to be useful for early screening and prognosis of lung cancer patients (Chen et al., 2017).

## Challenges in Clinical Application of QDs

Nanotechnology represents great potential for both basic cancer research and clinical application. However, compared with current conventional technologies, the novel QDs-based technologies have raised concerns of biosafety, reproducibility, and clinical reliability.

## Toxico-Genomics Implications

A major obstacle for integrating QDs into clinical application is the QD induced cytotoxicity. As QDs are composed of toxic metal atoms, therefore their ability to induce cytotoxicity through photon-induced free-radical formation and colloidal effects increases (Smith and Nie, 2009). The most commonly used QDs comprise of cadmium core which possibly is the potential and main cause of QD toxicity. Cytotoxicity may be further instigated through promoting free-radical formation as the core molecules are electronically active and are prone to photo and air oxidation. Emerging evidences also suggest that the cytotoxicity of free cadmium ions is linked to free-radical generation and not to cadmium release from QDs and may further lead to DNA damage in the presence or absence of light photon activation (Green and Howman, 2005; Cho et al., 2007). In addition to core material, a broader role of capping materials likes mercaptoacetic acid and trioctylphosphine oxide in the QD toxicity. Moreover, QD functionalization is an essential process for attachment of targeting different biological moieties and need to be investigated for their toxicity. Apart from toxicity, targeting ability of the material needs to be evaluated, as migration to the non-targeted site may lead to toxicity especially when QD function as a photosensitizing agent or drug carrier. Data for biostability of the coatings and their half-life *in vivo*, requires further analysis. All these queries are required to be answered before the clinical use of coated QD in human applications (Ghaderi et al., 2010). Many efficient attempts have been made to reduce the toxicity with preserved fluorescence properties of QDs. Preferring non-toxic and biodegradable nanomaterials as drug/gene carriers and finding substitutes of heavy metal based QDs could be one method for reducing toxicity. In addition, the use of non-toxic elements such as silica, zinc, sulfur, and copper may assist to solve the problem of heavy-metal toxicity. Graphene QDs due to their low toxicity have also emerged as promising alternatives in the field of cancer imaging. Nevertheless, development of core/shell QDs or encapsulated QDs by non-toxic materials could possibly be a promising attempt to reduce the toxicity and preserve a good fluorescence of the functional QDs.

The encapsulation of QDs through non-toxic materials like silica, nanogel, cross-linked dendrimers, nucleotide, and copolymer have showed remarkably reduced QD cytotoxicity (He et al., 2015).

## Design and Generation of Biocompatible Nanoparticles

Apart from toxicity, QDs are susceptible to non-specific organ uptake and RES scavenging due to their large size (15–30 nm) and short half-life in the systemic circulation, limiting their *in vivo* imaging and targeting applications. Various attempts have been made to overcome the *in vivo* limitations of QDs by attaching passivating molecules, such as PEG, and by controlling the overall charge of the particles to prevent their adsorption to the plasma proteins. An intriguing recent finding suggests a size threshold of 5–6 nm in diameter, below which the QDs cannot escape the liver and will be cleared through kidneys (Fang et al., 2017).

## Stability, Reproducibility, and Standardization

At the current stage, the clinical applicability of QD-based technologies is limited due to their reproducibility and comparability issues. The fluorescence quantum yields based on variable materials and surface chemistries will be different for different QDs obtained from various sources. Thus, the establishment and implementation of quality criteria for these materials of different functionalized QDs is the essential initial step (Fang et al., 2012). In order to translate QDs-based technologies working to clinical settings, it is necessary to train professional technicians to improve the familiarity and experience with QD-based tools as well as establish confidence in this technology with scientific and medical communities. Thus, it is of significant urgency to develop and establish the quality criteria or standardization for the labeling, imaging capture and followed quantitative analysis.

## Future Perspective

The increasing disease trend and limitations of traditional diagnostic modalities for lung cancer necessitate identifying novel markers capable of specific disease diagnosis at an early stage. There is convincing scientific evidence which suggests the potential role of miRNAs in initiation and progression of lung cancers. This led to the development of different detection technologies including qPCR and ISH capable to detect miRNA based in different types of samples. Both microarray and qRT-PCR platforms offer large-scale data and higher degree of accuracy but are only limited to the known miRNAs and lack adequate data validation. NGS may help to overcome these issues, but its higher cost is a major obstacle. An ideal miRNA-based point-of-care diagnosis should be capable to quickly detect disease-specific miRNA even at lower concentrations and should be reproducible with an appropriate multiplexing ability. Recent development has shown that the available miRNA detection strategies fulfill one or more requirements. In this regard, the potential use of QDs for cancer cell imaging and detection, have been widely appreciated. The availability of large emission spectrum and surface chemistries of QDs offer long-term stability, significantly higher sensitivity and multifunctional ability, making them versatile bio-probes. In addition, the use of non-toxic shells along with surface modification not only overcome their toxicity issues, but also increases the ability of these nanoshells to quickly identify cancer-associated molecular markers in a non-invasive mode. Although QDs are reported to efficiently conjugate with different biomolecules including DNA and RNA, their clinical utility is hampered by the scarcity of consistent methods and reproducible data. However, employing a systematic approach with appropriate control over their orientation, and avidity may certainly improve their ability. In addition, utilizing multifunctional QD-based analytical methods with immunohistochemistry or *in situ* nucleic acid hybridization in multiplexed format will be particularly significant. Importantly, much focus should be given toward the use of non-invasive or minimal invasive liquid biopsies-based detection methods as it provides the much clear picture of disease evolution over time.

## Author Contributions

RDS, RS, AB, RK, and RKS conducted the comprehensive literature review. RDS, RS, and RK wrote the manuscript. RT, KC, and IG helped in formulation of tables and figures and revised the article for important intellectual content. PM designed the concept of the manuscript, interpreted and analyzed the data. All authors read and approved the final manuscript.

## Conflict of Interest Statement

The authors declare that the research was conducted in the absence of any commercial or financial relationships that could be construed as a potential conflict of interest.

## Abbreviations

AGO: argonaute
BRET: bioluminescence resonance energy transfer
C9: component 9
ccf-miRNA: circulating cell-free miRNA
CEA: carcinoembryonic antigen
CL: chemiluminescence
CRET: chemiluminescence resonance energy transfer
CVD: chemical vapor deposition
CYFRA-21-1: cytokeratin fragment-21-1
DGCR-8: DiGeorge syndrome critical region gene 8
EMT: epithelial to mesenchymal transition
EXP-5: Exportin-5
FIB: focused ion beam
FRET: Förster resonance energy transfer
HDL: high-density lipoprotein
Hp β: Haptoglobin β Chain
IGFBP-2: insulin like growth factor binding protein-2
ISH: *in situ* hybridization
LDCT: low-dose computed tomography
LDL: low-density lipoprotein
LPCVD: low pressure CVD
MBE: molecular beam epitaxy
MDK: midkine
MOCVD: metal organic CVD
NMP1: nucleophosmin 1
NSCLC: non-small cell lung cancer
NSE: neuron-specific enolase
PECVD: plasma enhanced CVD
PKM2: M2-Pyruvate kinase
PVD: physical vapor deposition
QD: quantum dots
RISC: RNA induced silencing complex
SAA: serum amyloid A
SCLC: small cell lung cancer
TFPI: tissue factor pathway inhibitor
TIMP: tissue inhibitor of metalloproteinase

## References

[B1] Aberle D. R. Adams A. M. Berg C. D. Black W. C. Clapp J. D. Fagerstrom R. M. (2011). Reduced lung-cancer mortality with low-dose computed tomographic screening. *N. Engl. J. Med.* 365 395–409. 10.1056/NEJMoa1102873 21714641 PMC4356534

[B2] Adams B. D. Parsons C. Slack F. J. (2016). The tumor-suppressive and potential therapeutic functions of miR-34a in epithelial carcinomas. *Expert Opin. Ther. Targets* 20 737–753. 10.1517/14728222.2016.1114102 26652031 PMC4922263

[B3] Aharon A. Katzenell S. Tamari T. Brenner B. (2009). Microparticles bearing tissue factor and tissue factor pathway inhibitor in gestational vascular complications. *J. Thromb. Haemost.* 7 1047–1050. 10.1111/j.1538-7836.2009.03342.x 19320826

[B4] Ambros V. (2004). The functions of animal microRNAs. *Nature* 431 350–355. 10.1038/nature02871 15372042

[B5] An H. J. Lee Y. J. Hong S. A. Kim J. O. Lee K. Y. Kim Y. K. (2015). The prognostic role of tissue and serum MMP-1 and TIMP-1 expression in patients with non-small cell lung cancer. *Pathol. Res. Pract.* 212 357–364. 10.1016/j.prp.2015.11.014 26995105

[B6] Arroyo J. D. Chevillet J. R. Kroh E. M. Ruf I. K. Pritchard C. C. Gibson D. F. (2011). Argonaute2 complexes carry a population of circulating microRNAs independent of vesicles in human plasma. *Proc. Natl. Acad. Sci. U.S.A.* 108 5003–5008. 10.1073/pnas.1019055108 21383194 PMC3064324

[B7] Augustsson C. Hilden I. Petersen L. C. (2014). Inhibitory effects of LDL-associated tissue factor pathway inhibitor. *Thromb. Res.* 134 132–137. 10.1016/j.thromres.2014.03.043 24787991

[B8] Babin P. J. Gibbons G. F. (2009). The evolution of plasma cholesterol: direct utility or a “spandrel” of hepatic lipid metabolism? *Prog. Lipid Res.* 48 73–91. 10.1016/j.plipres.2008.11.002 19049814

[B9] Banerjee A. Grazon C. Nadal B. Pons T. Krishnan Y. Dubertret B. (2015). Fast, efficient, and stable conjugation of multiple DNA strands on colloidal quantum dots. *Bioconjug. Chem.* 26 1582–1589. 10.1021/acs.bioconjchem.5b00221 25992903

[B10] Beloglazova N. V. Foubert A. Gordienko A. Tessier M. D. Aubert T. Drijvers E. (2016). Sensitive QD@SiO2-based immunoassay for triplex determination of cereal-borne mycotoxins. *Talanta* 160 66–71. 10.1016/j.talanta.2016.05.015 27591588

[B11] Beloglazova N. V. Speranskaya E. S. Wu A. Wang Z. Sanders M. Goftman V. V. (2014). Novel multiplex fluorescent immunoassays based on quantum dot nanolabels for mycotoxins determination. *Biosens. Bioelectron.* 62 59–65. 10.1016/j.bios.2014.06.021 24976152

[B12] Bera D. Qian L. Sabui S. Santra S. Holloway P. (2008). Photoluminescence of ZnO quantum dots produced by a sol–gel process. *Opt. Mater.* 30 1233–1239. 10.1016/j.optmat.2007.06.001

[B13] Bhatnagar D. Kumar V. Kumar A. Kaur I. (2016). Graphene quantum dots FRET based sensor for early detection of heart attack in human. *Biosens. Bioelectron.* 79 495–499. 10.1016/j.bios.2015.12.083 26748366

[B14] Biaoxue R. Hua L. Wenlong G. Shuanying Y. (2016). Increased serum amyloid A as potential diagnostic marker for lung cancer: a meta-analysis based on nine studies. *BMC Cancer* 16:836. 10.1186/s12885-016-2882-0 27809798 PMC5093952

[B15] Bjerager M. Palshof T. Dahl R. Vedsted P. Olesen F. (2006). Delay in diagnosis of lung cancer in general practice. *Br. J. Gen. Pract.* 56 863–868. 17132354 PMC1927095

[B16] Blankenburg F. Hatz R. Nagel D. Ankerst D. Reinmiedl J. Gruber C. (2008). Preoperative CYFRA 21-1 and CEA as prognostic factors in patients with stage I non-small cell lung cancer: external validation of a prognostic score. *Tumour Biol.* 29 272–277. 10.1159/000152945 18781099

[B17] Boelens M. C. Wu T. J. Nabet B. Y. Xu B. Qiu Y. Yoon T. (2014). Exosome transfer from stromal to breast cancer cells regulates therapy resistance pathways. *Cell* 159 499–513. 10.1016/j.cell.2014.09.051 25417103 PMC4283810

[B18] Bommer G. T. Gerin I. Feng Y. Kaczorowski A. J. Kuick R. Love R. E. (2007). p53-mediated activation of miRNA34 candidate tumor-suppressor genes. *Curr. Biol.* 17 1298–1307. 10.1016/j.cub.2007.06.068 17656095

[B19] Borghei Y.-S. Hosseini M. Ganjali M. R. (2017). Fluorometric determination of microRNA via FRET between silver nanoclusters and CdTe quantum dots. *Microchim. Acta* 184 4713–4721.

[B20] Boyerinas B. Park S. M. Hau A. Murmann A. E. Peter M. E. (2010). The role of let-7 in cell differentiation and cancer. *Endocr. Relat. Cancer* 17 F19–F36. 10.1677/ERC-09-0184 19779035

[B21] Bray F. Ferlay J. Soerjomataram I. Siegel R. L. Torre L. A. Jemal A. (2018). Global cancer statistics 2018: GLOBOCAN estimates of incidence and mortality worldwide for 36 cancers in 185 countries. *CA Cancer J. Clin.* 68 394–424. 10.3322/caac.21492 30207593

[B22] Brenner D. J. (2012). Radiation and chest CT scans: are there problems? What should we do? *Chest* 142 549–550. 10.1378/chest.12-0490 22948569

[B23] Calin G. A. Sevignani C. Dumitru C. D. Hyslop T. Noch E. Yendamuri S. (2004). Human microRNA genes are frequently located at fragile sites and genomic regions involved in cancers. *Proc. Natl. Acad. Sci. U.S.A.* 101 2999–3004. 10.1073/pnas.0307323101 14973191 PMC365734

[B24] Catanzaro J. M. Guerriero J. L. Liu J. Ullman E. Sheshadri N. Chen J. J. (2011). Elevated expression of squamous cell carcinoma antigen (SCCA) is associated with human breast carcinoma. *PLoS One* 6:e19096. 10.1371/journal.pone.0019096 21526154 PMC3079753

[B25] Ceppi P. Mudduluru G. Kumarswamy R. Rapa I. Scagliotti G. V. Papotti M. (2010). Loss of miR-200c expression induces an aggressive, invasive, and chemoresistant phenotype in non-small cell lung cancer. *Mol. Cancer Res.* 8 1207–1216. 10.1158/1541-7786.MCR-10-0052 20696752

[B26] Chandan H. R. Schiffman J. D. Balakrishna R. G. (2018). Quantum dots as fluorescent probes: synthesis, surface chemistry, energy transfer mechanisms, and applications. *Sens. Actuators B* 258 1191–1214. 10.1016/j.snb.2017.11.189 22948544

[B27] Chen X. Ba Y. Ma L. Cai X. Yin Y. Wang K. (2008). Characterization of microRNAs in serum: a novel class of biomarkers for diagnosis of cancer and other diseases. *Cell Res.* 18 997–1006. 10.1038/cr.2008.282 18766170

[B28] Chen X. Hu Z. Wang W. Ba Y. Ma L. Zhang C. (2012). Identification of ten serum microRNAs from a genome-wide serum microRNA expression profile as novel noninvasive biomarkers for nonsmall cell lung cancer diagnosis. *Int. J. Cancer* 130 1620–1628. 10.1002/ijc.26177 21557218

[B29] Chen Z. Liang R. Guo X. Liang J. Deng Q. Li M. (2017). Simultaneous quantitation of cytokeratin-19 fragment and carcinoembryonic antigen in human serum via quantum dot-doped nanoparticles. *Biosens. Bioelectron.* 91 60–65. 10.1016/j.bios.2016.12.036 27988480

[B30] Cheng G. (2015). Circulating miRNAs: roles in cancer diagnosis, prognosis and therapy. *Adv. Drug Deliv. Rev.* 81 75–93. 10.1016/j.addr.2014.09.001 25220354

[B31] Chim S. S. Shing T. K. Hung E. C. Leung T. Y. Lau T. K. Chiu R. W. (2008). Detection and characterization of placental microRNAs in maternal plasma. *Clin. Chem.* 54 482–490. 10.1373/clinchem.2007.097972 18218722

[B32] Chin L. J. Slack F. J. (2008). A truth serum for cancer–microRNAs have major potential as cancer biomarkers. *Cell Res.* 18 983–984. 10.1038/cr.2008.290 18833286

[B33] Chironi G. N. Boulanger C. M. Simon A. Dignat-George F. Freyssinet J. M. Tedgui A. (2009). Endothelial microparticles in diseases. *Cell Tissue Res.* 335 143–151. 10.1007/s00441-008-0710-9 18989704

[B34] Cho S. J. Maysinger D. Jain M. RãDer B. Hackbarth S. Winnik F. O. M. (2007). Long-term exposure to CdTe quantum dots causes functional impairments in live cells. *Langmuir* 23 1974–1980. 10.1021/la060093j 17279683

[B35] Cho W. C. Yip T. T. Cheng W. W. Au J. S. (2010). Serum amyloid A is elevated in the serum of lung cancer patients with poor prognosis. *Br. J. Cancer* 102 1731–1735. 10.1038/sj.bjc.6605700 20502455 PMC2883701

[B36] Chong E. Z. Matthews D. R. Summers H. D. Njoh K. L. Errington R. J. Smith P. J. (2007). Development of FRET-based assays in the far-red using CdTe quantum dots. *J. Biomed. Biotechnol.* 2007:54169. 10.1155/2007/54169 18273410 PMC2217589

[B37] Collins L. G. Haines C. Perkel R. Enck R. E. (2007). Lung cancer: diagnosis and management. *Am. Fam. Physician* 75 56–63. 17225705

[B38] Cruz K. J. C. De Oliveira A. R. S. Morais J. B. S. Severo J. S. Marreiro D. D. N. (2017). Role of microRNAs on adipogenesis, chronic low-grade inflammation, and insulin resistance in obesity. *Nutrition* 35 28–35. 10.1016/j.nut.2016.10.003 28241987

[B39] Cui E. H. Li H. J. Hua F. Wang B. Mao W. Feng X. R. (2013). Serum microRNA 125b as a diagnostic or prognostic biomarker for advanced NSCLC patients receiving cisplatin-based chemotherapy. *Acta Pharmacol. Sin.* 34 309–313. 10.1038/aps.2012.125 22983388 PMC4011618

[B40] Damarla K. Bharmoria P. Rao K. S. Gehlot P. S. Kumar A. (2016). Illuminating microemulsions: ionic liquid-CdS quantum dots hybrid materials as potential white light harvesting systems. *Chem. Commun.* 52 6320–6323. 10.1039/C6CC02133F 27087045

[B41] Dave B. J. Hess M. M. Pickering D. L. Zaleski D. H. Pfeifer A. L. Weisenburger D. D. (1999). Rearrangements of chromosome band 1p36 in non-Hodgkin’s lymphoma. *Clin. Cancer Res.* 5 1401–1409. 10389925

[B42] de Paula R. R. Marinho F. V. Fahel J. S. Oliveira S. C. (2017). Contribution of intercellular adhesion molecule 1 (ICAM-1) to control *Mycobacterium avium* infection. *Microbes Infect.* 19 527–535. 10.1016/j.micinf.2017.09.005 28943322

[B43] Dhawan S. Dhawan T. Vedeshwar A. G. (2014). Growth of Nb2O5 quantum dots by physical vapor deposition. *Mater. Lett.* 126 32–35. 10.1016/j.matlet.2014.03.107

[B44] Dirks R. M. Pierce N. A. (2004). Triggered amplification by hybridization chain reaction. *Proc. Natl. Acad. Sci U.S.A.* 101 15275–15278. 10.1073/pnas.0407024101 15492210 PMC524468

[B45] Draghici S. Khatri P. Eklund A. C. Szallasi Z. (2006). Reliability and reproducibility issues in DNA microarray measurements. *Trends Genet.* 22 101–109. 10.1016/j.tig.2005.12.005 16380191 PMC2386979

[B46] Dudkina N. V. Spicer B. A. Reboul C. F. Conroy P. J. Lukoyanova N. Elmlund H. (2016). Structure of the poly-C9 component of the complement membrane attack complex. *Nat. Commun.* 7:10588. 10.1038/ncomms10588 26841934 PMC4742998

[B47] Eklund K. K. Niemi K. Kovanen P. T. (2012). Immune functions of serum amyloid A. *Crit. Rev. Immunol.* 32 335–348. 23237509 10.1615/critrevimmunol.v32.i4.40

[B48] El-Hefnawy T. Raja S. Kelly L. Bigbee W. L. Kirkwood J. M. Luketich J. D. (2004). Characterization of amplifiable, circulating RNA in plasma and its potential as a tool for cancer diagnostics. *Clin. Chem.* 50 564–573. 10.1373/clinchem.2003.028506 14718398

[B49] Eminaga S. Christodoulou D. C. Vigneault F. Church G. M. Seidman J. G. (2013). Quantification of microRNA expression with next-generation sequencing. *Curr. Protoc. Mol. Biol.* 103 4.17.1–4.17.14. 10.1002/0471142727.mb0417s103 23821442 PMC4138881

[B50] Esquela-Kerscher A. Slack F. J. (2006). Oncomirs - microRNAs with a role in cancer. *Nat. Rev. Cancer* 6 259–269. 10.1038/nrc1840 16557279

[B51] Fabbri M. Paone A. Calore F. Galli R. Gaudio E. Santhanam R. (2012). MicroRNAs bind to Toll-like receptors to induce prometastatic inflammatory response. *Proc. Natl. Acad. Sci. U.S.A.* 109 E2110–E2116. 10.1073/pnas.1209414109 22753494 PMC3412003

[B52] Fan L. Qi H. Teng J. Su B. Chen H. Wang C. (2016). Identification of serum miRNAs by nano-quantum dots microarray as diagnostic biomarkers for early detection of non-small cell lung cancer. *Tumour Biol.* 37 7777–7784. 10.1007/s13277-015-4608-3 26695145

[B53] Fang M. Chen M. Liu L. Li Y. (2017). Applications of quantum dots in cancer detection and diagnosis: a review. *J. Biomed. Nanotechnol.* 13 1–16. 29372982 10.1166/jbn.2017.2334

[B54] Fang M. Peng C. W. Pang D. W. Li Y. (2012). Quantum dots for cancer research: current status, remaining issues, and future perspectives. *Cancer Biol. Med.* 9 151–163. 10.7497/j.issn.2095-3941.2012.03.001 23691472 PMC3643664

[B55] Fei X. Wang H. Yuan W. Wo M. Jiang L. (2017). Tissue factor pathway inhibitor-1 is a valuable marker for the prediction of deep venous thrombosis and tumor metastasis in patients with lung cancer. *Biomed. Res. Int.* 2017:8983763. 10.1155/2017/8983763 28246607 PMC5299162

[B56] Fernandez-Mercado M. Manterola L. Lawrie C. H. (2015). MicroRNAs in lymphoma: regulatory role and biomarker potential. *Curr. Genomics* 16 349–358. 10.2174/1389202916666150707160147 27047255 PMC4763973

[B57] Foss K. M. Sima C. Ugolini D. Neri M. Allen K. E. Weiss G. J. (2011). miR-1254 and miR-574-5p: serum-based microRNA biomarkers for early-stage non-small cell lung cancer. *J. Thorac. Oncol.* 6 482–488. 10.1097/JTO.0b013e318208c785 21258252

[B58] Franchina T. Amodeo V. Bronte G. Savio G. Ricciardi G. R. Picciotto M. (2014). Circulating miR-22, miR-24 and miR-34a as novel predictive biomarkers to pemetrexed-based chemotherapy in advanced non-small cell lung cancer. *J. Cell. Physiol.* 229 97–99. 10.1002/jcp.24422 23794259

[B59] Freeman R. Liu X. Willner I. (2011). Chemiluminescent and Chemiluminescence Resonance Energy Transfer (CRET) Detection of DNA, metal ions, and aptamer–substrate complexes using Hemin/G-Quadruplexes and CdSe/ZnS Quantum Dots. *J. Am. Chem. Soc.* 133 11597–11604. 10.1021/ja202639m 21678959

[B60] Fujita N. Kagara N. Yamamoto N. Shimazu K. Shimomura A. Shimoda M. (2014). Methylated DNA and high total DNA levels in the serum of patients with breast cancer following neoadjuvant chemotherapy are predictive of a poor prognosis. *Oncol. Lett.* 8 397–403. 10.3892/ol.2014.2068 24959284 PMC4063626

[B61] Fujita Y. Kosaka N. Araya J. Kuwano K. Ochiya T. (2015). Extracellular vesicles in lung microenvironment and pathogenesis. *Trends Mol. Med.* 21 533–542. 10.1016/j.molmed.2015.07.004 26231094

[B62] Gallach S. Jantus-Lewintre E. Calabuig-Farinas S. Montaner D. Alonso S. Sirera R. (2017). MicroRNA profiling associated with non-small cell lung cancer: next generation sequencing detection, experimental validation, and prognostic value. *Oncotarget* 8 56143–56157. 10.18632/oncotarget.18603 28915579 PMC5593550

[B63] Gallo A. Tandon M. Alevizos I. Illei G. G. (2012). The majority of microRNAs detectable in serum and saliva is concentrated in exosomes. *PLoS One* 7:e30679. 10.1371/journal.pone.0030679 22427800 PMC3302865

[B64] Garofalo M. Di Leva G. Romano G. Nuovo G. Suh S. S. Ngankeu A. (2009). miR-221&222 regulate TRAIL resistance and enhance tumorigenicity through PTEN and TIMP3 downregulation. *Cancer Cell* 16 498–509. 10.1016/j.ccr.2009.10.014 19962668 PMC2796583

[B65] Garofalo M. Jeon Y. J. Nuovo G. J. Middleton J. Secchiero P. Joshi P. (2013). MiR-34a/c-Dependent PDGFR-alpha/beta downregulation inhibits tumorigenesis and enhances TRAIL-induced apoptosis in lung cancer. *PLoS One* 8:e67581. 10.1371/journal.pone.0067581 23805317 PMC3689725

[B66] Gazdar A. F. Minna J. D. (2008). Deregulated EGFR signaling during lung cancer progression: mutations, amplicons, and autocrine loops. *Cancer Prev. Res.* 1 156–160. 10.1158/1940-6207.CAPR-08-0080 19138950 PMC2849648

[B67] Ghaderi S. Ramesh B. Seifalian A. (2010). Fluorescence nanoparticles “quantum dots” as drug delivery system and their toxicity: a review. *J. Drug Target* 19 475–486. 10.3109/1061186X.2010.526227 20964619

[B68] Gong Y.-J. Zhang X.-B. Mao G.-J. Su L. Meng H.-M. Tan W. (2016). A unique approach toward near-infrared fluorescent probes for bioimaging with remarkably enhanced contrast. *Chem. Sci.* 7 2275–2285. 10.1039/C5SC04014K 29910917 PMC5977507

[B69] Goulart B. H. Bensink M. E. Mummy D. G. Ramsey S. D. (2012). Lung cancer screening with low-dose computed tomography: costs, national expenditures, and cost-effectiveness. *J. Natl. Compr. Canc. Netw.* 10 267–275. 22308519 10.6004/jnccn.2012.0023

[B70] Green M. Howman E. (2005). Semiconductor quantum dots and free radical induced DNA nicking. *Chem. Commun.* 5 121–123. 10.1039/b413175d 15614393

[B71] Gregory P. A. Bert A. G. Paterson E. L. Barry S. C. Tsykin A. Farshid G. (2008). The miR-200 family and miR-205 regulate epithelial to mesenchymal transition by targeting ZEB1 and SIP1. *Nat. Cell Biol.* 10 593–601. 10.1038/ncb1722 18376396

[B72] Haffner M. C. Mosbruger T. Esopi D. M. Fedor H. Heaphy C. M. Walker D. A. (2013). Tracking the clonal origin of lethal prostate cancer. *J. Clin. Invest.* 123 4918–4922. 10.1172/JCI70354 24135135 PMC3809798

[B73] Halvorsen A. R. Bjaanaes M. Leblanc M. Holm A. M. Bolstad N. Rubio L. (2016). A unique set of 6 circulating microRNAs for early detection of non-small cell lung cancer. *Oncotarget* 7 37250–37259. 10.18632/oncotarget.9363 27191990 PMC5095073

[B74] Hammarstrom S. (1999). The carcinoembryonic antigen (CEA) family: structures, suggested functions and expression in normal and malignant tissues. *Semin. Cancer Biol.* 9 67–81. 10.1006/scbi.1998.0119 10202129

[B75] Hanagiri T. Sugaya M. Takenaka M. Oka S. Baba T. Shigematsu Y. (2011). Preoperative CYFRA 21-1 and CEA as prognostic factors in patients with stage I non-small cell lung cancer. *Lung Cancer* 74 112–117. 10.18632/oncotarget.9363 21397974

[B76] Hanke M. Hoefig K. Merz H. Feller A. C. Kausch I. Jocham D. (2010). A robust methodology to study urine microRNA as tumor marker: microRNA-126 and microRNA-182 are related to urinary bladder cancer. *Urol. Oncol.* 28 655–661. 10.1016/j.urolonc.2009.01.027 19375957

[B77] Hayashita Y. Osada H. Tatematsu Y. Yamada H. Yanagisawa K. Tomida S. (2005). A polycistronic microRNA cluster, miR-17-92, is overexpressed in human lung cancers and enhances cell proliferation. *Cancer Res.* 65 9628–9632. 10.1158/0008-5472.CAN-05-2352 16266980

[B78] He C. L. Bian Y. Y. Xue Y. Liu Z. X. Zhou K. Q. Yao C. F. (2016). Pyruvate Kinase M2 Activates mTORC1 by Phosphorylating AKT1S1. *Sci. Rep.* 6:21524. 10.1038/srep21524 26876154 PMC4753445

[B79] He D. Wang D. Quan W. Yu C. (2015). Functional quantum dots for promising cancer diagnosis and therapy. *J. Nanomed. Nanosurg.* 1. 10.16966/2470-3206.103

[B80] Heegaard N. H. Schetter A. J. Welsh J. A. Yoneda M. Bowman E. D. Harris C. C. (2012). Circulating micro-RNA expression profiles in early stage non-small cell lung cancer. *Int. J. Cancer* 130 1378–1386. 10.1002/ijc.26153 21544802 PMC3259258

[B81] Heneghan H. M. Miller N. Lowery A. J. Sweeney K. J. Newell J. Kerin M. J. (2010). Circulating microRNAs as novel minimally invasive biomarkers for breast cancer. *Ann. Surg.* 251 499–505. 10.1097/SLA.0b013e3181cc939f 20134314

[B82] Hermeking H. (2010). The miR-34 family in cancer and apoptosis. *Cell Death Differ.* 17 193–199. 10.1038/cdd.2009.56 19461653

[B83] Howlader N. Noone A. M. Krapcho M. Garshell J. Neyman N. Altekruse S. (2013). *SEER Cancer Statistics Review, 1975-2010.[Based on the November 2012 SEER data submission, posted to the SEER web site, April 2013.]*. Bethesda, MD: National Cancer Institute.

[B84] Hsu C. Y. Chen C. W. Yu H.-P. Lin Y.-F. Lai P. S. (2012). Bioluminescence resonance energy transfer using luciferase-immobilized quantum dots for self-illuminated photodynamic therapy. *Biomaterials* 34 1204–1212. 10.1016/j.biomaterials.2012.08.044 23069718

[B85] Huang J. Wu J. Li Y. Li X. Yang T. Yang Q. (2014). Deregulation of serum microRNA expression is associated with cigarette smoking and lung cancer. *Biomed. Res. Int.* 2014:364316. 10.1155/2014/364316 25386559 PMC4217347

[B86] Huang X. Li L. Qian H. Dong C. Ren J. (2006). A resonance energy transfer between chemiluminescent donors and luminescent quantum-dots as acceptors (CRET). *Angew. Chem. Int. Ed.* 45 5140–5143. 16826612 10.1002/anie.200601196

[B87] Hutvagner G. Mclachlan J. Pasquinelli A. E. Balint E. Tuschl T. Zamore P. D. (2001). A cellular function for the RNA-interference enzyme Dicer in the maturation of the let-7 small temporal RNA. *Science* 293 834–838. 10.1126/science.1062961 11452083

[B88] Ji X. Takahashi R. Hiura Y. Hirokawa G. Fukushima Y. Iwai N. (2009). Plasma miR-208 as a biomarker of myocardial injury. *Clin. Chem.* 55 1944–1949. 10.1373/clinchem.2009.125310 19696117

[B89] Jing X. Cui X. Liang H. Hao C. Han C. (2017). Diagnostic accuracy of ELISA for detecting serum Midkine in cancer patients. *PLoS One* 12:e0180511. 10.1371/journal.pone.0180511 28686647 PMC5501560

[B90] Johnson S. M. Grosshans H. Shingara J. Byrom M. Jarvis R. Cheng A. (2005). RAS is regulated by the let-7 microRNA family. *Cell* 120 635–647. 10.1016/j.cell.2005.01.014 15766527

[B91] Jumper C. Cobos E. Lox C. (2004). Determination of the serum matrix metalloproteinase-9 (MMP-9) and tissue inhibitor of matrix metalloproteinase-1 (TIMP-1) in patients with either advanced small-cell lung cancer or non-small-cell lung cancer prior to treatment. *Respir. Med.* 98 173–177. 14971882 10.1016/j.rmed.2003.08.014

[B92] Jung E. J. Calin G. A. (2010). The Meaning of 21 in the MicroRNA world: perfection rather than destruction? *Cancer Cell* 18 203–205. 10.1016/j.ccr.2010.08.015 20832748

[B93] Kakkar R. Malik P. Gulia S. (2013). Quantum dots for diagnosis of cancers. *Adv. Mat. Lett.* 4 811–822. 10.5185/amlett.2013.34372013

[B94] Kaller M. Liffers S. T. Oeljeklaus S. Kuhlmann K. Roh S. Hoffmann R. (2011). Genome-wide characterization of miR-34a induced changes in protein and mRNA expression by a combined pulsed SILAC and microarray analysis. *Mol. Cell. Proteomics* 10:M111.010462. 10.1074/mcp.M111.010462 21566225 PMC3149097

[B95] Kamkaew A. Sun H. England C. G. Cheng L. Liu Z. Cai W. (2016). Quantum dot-NanoLuc bioluminescence resonance energy transfer enables tumor imaging and lymph node mapping in vivo. *Chem. Commun.* 52 6997–7000. 10.1039/c6cc02764d 27157466 PMC4912020

[B96] Kang S. M. Sung H. J. Ahn J. M. Park J. Y. Lee S. Y. Park C. S. (2011). The Haptoglobin beta chain as a supportive biomarker for human lung cancers. *Mol. Biosyst.* 7 1167–1175. 10.1039/c0mb00242a 21253648

[B97] Karmakar R. (2015). Quantum Dots and it method of preparations - revisited. *Prajnan O Sadhona* 2 116–142.

[B98] Kasinski A. L. Slack F. J. (2012). miRNA-34 prevents cancer initiation and progression in a therapeutically resistant K-ras and p53-induced mouse model of lung adenocarcinoma. *Cancer Res.* 72 5576–5587. 10.1158/0008-5472.CAN-12-2001 22964582 PMC3488137

[B99] Khakbaz F. Mahani M. (2017). Micro-RNA detection based on fluorescence resonance energy transfer of DNA-carbon quantum dots probes. *Anal. Biochem.* 523 32–38. 10.1016/j.ab.2017.01.025 28159568

[B100] Kim S. I. Shin D. Choi T. H. Lee J. C. Cheon G. J. Kim K. Y. (2007). Systemic and specific delivery of small interfering RNAs to the liver mediated by apolipoprotein A-I. *Mol. Ther.* 15 1145–1152. 10.1038/sj.mt.6300168 17440441

[B101] Kogure T. Lin W. L. Yan I. K. Braconi C. Patel T. (2011). Intercellular nanovesicle-mediated microRNA transfer: a mechanism of environmental modulation of hepatocellular cancer cell growth. *Hepatology* 54 1237–1248. 10.1002/hep.24504 21721029 PMC3310362

[B102] Koleva R. I. Conley B. A. Romero D. Riley K. S. Marto J. A. Lux A. (2006). Endoglin structure and function: determinants of endoglin phosphorylation by transforming growth factor-beta receptors. *J. Biol. Chem.* 281 25110–25123. 10.1074/jbc.M601288200 16785228

[B103] Kopczynska E. Dancewicz M. Kowalewski J. Makarewicz R. Kardymowicz H. Kaczmarczyk A. (2012). Influence of surgical resection on plasma endoglin (CD105) level in nonsmall cell lung cancer patients. *Exp. Oncol.* 34 53–56. 22453150

[B104] Korpal M. Lee E. S. Hu G. Kang Y. (2008). The miR-200 family inhibits epithelial-mesenchymal transition and cancer cell migration by direct targeting of E-cadherin transcriptional repressors ZEB1 and ZEB2. *J. Biol. Chem.* 283 14910–14914. 10.1074/jbc.C800074200 18411277 PMC3258899

[B105] Koscianska E. Baev V. Skreka K. Oikonomaki K. Rusinov V. Tabler M. (2007). Prediction and preliminary validation of oncogene regulation by miRNAs. *BMC Mol. Biol.* 8:79. 10.1186/1471-2199-8-79 17877811 PMC2096627

[B106] Kowalik M. K. Martyniak M. Rekawiecki R. Kotwica J. (2016). Expression and immunolocalization of membrane progesterone receptors in the bovine oviduct. *Domest. Anim. Endocrinol.* 55 83–96. 10.1016/j.domaniend.2015.12.001 26774557

[B107] Kuespert K. Pils S. Hauck C. R. (2006). CEACAMs: their role in physiology and pathophysiology. *Curr. Opin. Cell Biol.* 18 565–571. 10.1016/j.ceb.2006.08.008 16919437 PMC7127089

[B108] Kulpa J. Wojcik E. Reinfuss M. Kolodziejski L. (2002). Carcinoembryonic antigen, squamous cell carcinoma antigen, CYFRA 21-1, and neuron-specific enolase in squamous cell lung cancer patients. *Clin. Chem.* 48 1931–1937. 12406978

[B109] Larrea E. Sole C. Manterola L. Goicoechea I. Armesto M. Arestin M. (2016). New concepts in cancer biomarkers: circulating miRNAs in Liquid Biopsies. *Int. J. Mol. Sci.* 17:E627. 10.3390/ijms17050627 27128908 PMC4881453

[B110] Laterza O. F. Lim L. Garrett-Engele P. W. Vlasakova K. Muniappa N. Tanaka W. K. (2009). Plasma MicroRNAs as sensitive and specific biomarkers of tissue injury. *Clin. Chem.* 55 1977–1983. 10.1373/clinchem.2009.131797 19745058

[B111] Lawrie C. H. Gal S. Dunlop H. M. Pushkaran B. Liggins A. P. Pulford K. (2008). Detection of elevated levels of tumour-associated microRNAs in serum of patients with diffuse large B-cell lymphoma. *Br. J. Haematol.* 141 672–675. 10.1111/j.1365-2141.2008.07077.x 18318758

[B112] Lebrin F. Goumans M. J. Jonker L. Carvalho R. L. Valdimarsdottir G. Thorikay M. (2004). Endoglin promotes endothelial cell proliferation and TGF-beta/ALK1 signal transduction. *EMBO J.* 23 4018–4028. 10.1038/sj.emboj.7600386 15385967 PMC524335

[B113] Lee Y. Ahn C. Han J. Choi H. Kim J. Yim J. (2003). The nuclear RNase III Drosha initiates microRNA processing. *Nature* 425 415–419. 10.1038/nature01957 14508493

[B114] Lee Y. El Andaloussi S. Wood M. J. (2012). Exosomes and microvesicles: extracellular vesicles for genetic information transfer and gene therapy. *Hum. Mol. Genet.* 21 R125–R134. 10.1093/hmg/dds317 22872698

[B115] Lee Y. S. Dutta A. (2007). The tumor suppressor microRNA let-7 represses the HMGA2 oncogene. *Genes Dev.* 21 1025–1030. 10.1101/gad.1540407 17437991 PMC1855228

[B116] Li J. Wang Y. Xue S. Sun J. Zhang W. Hu P. (2016). Effective combination treatment of lung cancer cells by single vehicular delivery of siRNA and different anticancer drugs. *Int. J. Nanomed.* 11 4609–4624. 10.2147/IJN.S107345 27695321 PMC5028086

[B117] Li Y. Choi P. S. Casey S. C. Dill D. L. Felsher D. W. (2014). MYC through miR-17-92 suppresses specific target genes to maintain survival, autonomous proliferation, and a neoplastic state. *Cancer Cell* 26 262–272. 10.1016/j.ccr.2014.06.014 25117713 PMC4191901

[B118] Li Z. Xu Y. Wang Q. Xie C. Liu Y. Tu Z. (2017). Tissue factor pathway inhibitor-2 induced hepatocellular carcinoma cell differentiation. *Saudi J. Biol. Sci.* 24 95–102. 10.1016/j.sjbs.2016.09.003 28053577 PMC5199000

[B119] Liu L. Wu S. Jing F. Zhou H. Jia C. Li G. (2016). Bead-based microarray immunoassay for lung cancer biomarkers using quantum dots as labels. *Biosens. Bioelectron.* 80 300–306. 10.1016/j.bios.2016.01.084 26852198

[B120] Liu W. Yang Y. Liu Y. Lu X. Guo S. Wu M. (2016). Exogenous kallikrein protects against diabetic nephropathy. *Kidney Int.* 90 1023–1036. 10.1016/j.kint.2016.06.018 27546607

[B121] Lose F. Thompson P. J. Duffy D. Stewart G. A. Kedda M. A. (2005). A novel tissue inhibitor of metalloproteinase-1 (TIMP-1) polymorphism associated with asthma in Australian women. *Thorax* 60 623–628. 10.1136/thx.2004.026930 16061701 PMC1747496

[B122] Louhelainen N. Stark H. Mazur W. Rytila P. Djukanovic R. Kinnula V. L. (2010). Elevation of sputum matrix metalloproteinase-9 persists up to 6 months after smoking cessation: a research study. *BMC Pulm. Med.* 10:13. 10.1186/1471-2466-10-13 20226090 PMC2841651

[B123] Lund E. Guttinger S. Calado A. Dahlberg J. E. Kutay U. (2004). Nuclear export of microRNA precursors. *Science* 303 95–98. 10.1126/science.1090599 14631048

[B124] Luo X. Guo B. Wang L. Deng F. Qi R. Luo S. (2014). Synthesis of magnetic ion-imprinted fluorescent CdTe quantum dots by chemical etching and their visualization application for selective removal of Cd(II) from water. *Colloids Surf. A Physicochem. Eng. Asp.* 462 186–193. 10.1016/j.colsurfa.2014.09.012

[B125] Ma E. Fu Y. Garvey W. T. (2018). Relationship of circulating miRNAs with insulin sensitivity and associated metabolic risk factors in humans. *Metab. Syndr. Relat. Disord.* 16 82–89. 10.1089/met.2017.0101 29360415 PMC5833250

[B126] Ma J. Mannoor K. Gao L. Tan A. Guarnera M. A. Zhan M. (2014). Characterization of microRNA transcriptome in lung cancer by next-generation deep sequencing. *Mol. Oncol.* 8 1208–1219. 10.1016/j.molonc.2014.03.019 24785186 PMC4198444

[B127] Ma Y. Bai Y. Mao H. Hong Q. Yang D. Zhang H. (2016). A panel of promoter methylation markers for invasive and noninvasive early detection of NSCLC using a quantum dots-based FRET approach. *Biosens. Bioelectron.* 85 641–648. 10.1016/j.bios.2016.05.067 27240011

[B128] Ma Y. Zhang H. Liu F. Wu Z. Lu S. Jin Q. (2015). Highly sensitive detection of DNA methylation levels by using a quantum dot-based FRET method. *Nanoscale* 7 17547–17555. 10.1039/c5nr04956c 26446775

[B129] Mackillop W. J. (2007). Killing time: the consequences of delays in radiotherapy. *Radiother. Oncol.* 84 1–4. 10.1016/j.radonc.2007.05.006 17574695

[B130] Maeda N. Ichihara-Tanaka K. Kimura T. Kadomatsu K. Muramatsu T. Noda M. (1999). A receptor-like protein-tyrosine phosphatase PTPzeta/RPTPbeta binds a heparin-binding growth factor midkine. Involvement of arginine 78 of midkine in the high affinity binding to PTPzeta. *J. Biol. Chem.* 274 12474–12479. 10212223 10.1074/jbc.274.18.12474

[B131] Mandke P. Wyatt N. Fraser J. Bates B. Berberich S. J. Markey M. P. (2012). MicroRNA-34a modulates MDM4 expression via a target site in the open reading frame. *PLoS One* 7:e42034. 10.1371/journal.pone.0042034 22870278 PMC3411609

[B132] Mao L. Li J. Chen W. X. Cai Y. Q. Yu D. D. Zhong S. L. (2016). Exosomes decrease sensitivity of breast cancer cells to adriamycin by delivering microRNAs. *Tumour Biol.* 37 5247–5256. 10.1007/s13277-015-4402-2 26555545

[B133] Mardente S. Mari E. Massimi I. Fico F. Faggioni A. Pulcinelli F. (2015). HMGB1-induced cross talk between PTEN and miRs 221/222 in Thyroid Cancer. *Biomed. Res. Int.* 2015:512027. 10.1155/2015/512027 26106610 PMC4461734

[B134] Margulies M. Egholm M. Altman W. E. Attiya S. Bader J. S. Bemben L. A. (2005). Genome sequencing in microfabricated high-density picolitre reactors. *Nature* 437 376–380. 10.1038/nature03959 16056220 PMC1464427

[B135] Marzolla V. Armani A. Mammi C. Moss M. E. Pagliarini V. Pontecorvo L. (2017). Essential role of ICAM-1 in aldosterone-induced atherosclerosis. *Int. J. Cardiol.* 232 233–242. 10.1016/j.ijcard.2017.01.013 28089144 PMC5890338

[B136] Matsubara H. Takeuchi T. Nishikawa E. Yanagisawa K. Hayashita Y. Ebi H. (2007). Apoptosis induction by antisense oligonucleotides against miR-17-5p and miR-20a in lung cancers overexpressing miR-17-92. *Oncogene* 26 6099–6105. 10.1038/sj.onc.1210425 17384677

[B137] McCunney R. J. Li J. (2014). Radiation risks in lung cancer screening programs. *Chest* 145 618–624. 10.1378/chest.13-1420 27845636

[B138] Mishra P. Bhat B. R. (2018). Synthesis and characterization of graphene quantum dots and their size reduction using swift heavy ion beam. *Radiat. Effects Defects Solids* 173 232–238. 10.1080/10420150.2018.1424850

[B139] Mitchell P. S. Parkin R. K. Kroh E. M. Fritz B. R. Wyman S. K. Pogosova-Agadjanyan E. L. (2008). Circulating microRNAs as stable blood-based markers for cancer detection. *Proc. Natl. Acad. Sci. U.S.A.* 105 10513–10518. 10.1073/pnas.0804549105 18663219 PMC2492472

[B140] Mittelbrunn M. Gutierrez-Vazquez C. Villarroya-Beltri C. Gonzalez S. Sanchez-Cabo F. Gonzalez M. A. (2011). Unidirectional transfer of microRNA-loaded exosomes from T cells to antigen-presenting cells. *Nat. Commun.* 2:282. 10.1038/ncomms1285 21505438 PMC3104548

[B141] Montecalvo A. Larregina A. T. Shufesky W. J. Stolz D. B. Sullivan M. L. Karlsson J. M. (2012). Mechanism of transfer of functional microRNAs between mouse dendritic cells via exosomes. *Blood* 119 756–766. 10.1182/blood-2011-02-338004 22031862 PMC3265200

[B142] Murakami A. Fukushima C. Yositomi K. Sueoka K. Nawata S. Fujimoto M. (2010). Tumor-related protein, the squamous cell carcinoma antigen binds to the intracellular protein carbonyl reductase. *Int. J. Oncol.* 36 1395–1400. 20428762 10.3892/ijo_00000624

[B143] Muralidharan-Chari V. Clancy J. W. Sedgwick A. D’souza-Schorey C. (2010). Microvesicles: mediators of extracellular communication during cancer progression. *J. Cell Sci.* 123 1603–1611. 10.1242/jcs.064386 20445011 PMC2864708

[B144] Narayanasamy A. Ahn J. M. Sung H. J. Kong D. H. Ha K. S. Lee S. Y. (2011). Fucosylated glycoproteomic approach to identify a complement component 9 associated with squamous cell lung cancer (SQLC). *J. Proteomics* 74 2948–2958. 10.1016/j.jprot.2011.07.019 21840429

[B145] Noferesti S. S. Sohel M. M. Hoelker M. Salilew-Wondim D. Tholen E. Looft C. (2015). Controlled ovarian hyperstimulation induced changes in the expression of circulatory miRNA in bovine follicular fluid and blood plasma. *J. Ovarian Res.* 8:81. 10.1186/s13048-015-0208-5 26645573 PMC4673782

[B146] Nomoto S. Haruki N. Tatematsu Y. Konishi H. Mitsudomi T. Takahashi T. (2000). Frequent allelic imbalance suggests involvement of a tumor suppressor gene at 1p36 in the pathogenesis of human lung cancers. *Genes Chromosomes Cancer* 28 342–346. 10862041 10.1002/1098-2264(200007)28:3<342::aid-gcc13>3.0.co;2-a

[B147] Oh H. J. Park H. Y. Kim K. H. Park C. K. Shin H. J. Lim J. H. (2016). Progastrin-releasing peptide as a diagnostic and therapeutic biomarker of small cell lung cancer. *J. Thorac. Dis.* 8 2530–2537. 10.21037/jtd.2016.08.72 27747005 PMC5059255

[B148] Ohshima K. Inoue K. Fujiwara A. Hatakeyama K. Kanto K. Watanabe Y. (2010). Let-7 microRNA family is selectively secreted into the extracellular environment via exosomes in a metastatic gastric cancer cell line. *PLoS One* 5:e13247. 10.1371/journal.pone.0013247 20949044 PMC2951912

[B149] Okamura K. Takayama K. Izumi M. Harada T. Furuyama K. Nakanishi Y. (2013). Diagnostic value of CEA and CYFRA 21-1 tumor markers in primary lung cancer. *Lung Cancer* 80 45–49. 10.1016/j.lungcan.2013.01.002 23352032

[B150] Oremek G. M. Sapoutzis N. (2003). Pro-gastrin-releasing peptide (Pro-GRP), a tumor marker for small cell lung cancer. *Anticancer Res.* 23 895–898. 12820319

[B151] Osada H. Takahashi T. (2011). let-7 and miR-17-92: small-sized major players in lung cancer development. *Cancer Sci.* 102 9–17. 10.1111/j.1349-7006.2010.01707.x 20735434

[B152] Ottaiano T. F. Andrade S. S. De Oliveira C. Silva M. C. C. Buri M. V. Juliano M. A. (2017). Plasma kallikrein enhances platelet aggregation response by subthreshold doses of ADP. *Biochimie* 135 72–81. 10.1016/j.biochi.2017.01.010 28115185 PMC5346445

[B153] Pan J. Song G. Chen D. Li Y. Liu S. Hu S. (2017). Identification of serological biomarkers for early diagnosis of lung cancer using a protein array-based approach. *Mol. Cell. Proteomics* 16 2069–2078. 10.1074/mcp.RA117.000212 29021294 PMC5724172

[B154] Park N. J. Zhou H. Elashoff D. Henson B. S. Kastratovic D. A. Abemayor E. (2009). Salivary microRNA: discovery, characterization, and clinical utility for oral cancer detection. *Clin. Cancer Res.* 15 5473–5477. 10.1158/1078-0432.CCR-09-0736 19706812 PMC2752355

[B155] Park S. M. Gaur A. B. Lengyel E. Peter M. E. (2008). The miR-200 family determines the epithelial phenotype of cancer cells by targeting the E-cadherin repressors ZEB1 and ZEB2. *Genes Dev.* 22 894–907. 10.1101/gad.1640608 18381893 PMC2279201

[B156] Peluso J. J. Romak J. Liu X. (2008). Progesterone receptor membrane component-1 (PGRMC1) is the mediator of progesterone’s antiapoptotic action in spontaneously immortalized granulosa cells as revealed by PGRMC1 small interfering ribonucleic acid treatment and functional analysis of PGRMC1 mutations. *Endocrinology* 149 534–543. 10.1210/en.2007-1050 17991724 PMC2219306

[B157] Pfleger K. D. Eidne K. A. (2006). Illuminating insights into protein-protein interactions using bioluminescence resonance energy transfer (BRET). *Nat. Methods* 3 165–174. 10.1038/nmeth841 16489332

[B158] Polticelli F. Bocedi A. Minervini G. Ascenzi P. (2008). Human haptoglobin structure and function–a molecular modelling study. *FEBS J.* 275 5648–5656. 10.1111/j.1742-4658.2008.06690.x 18959750

[B159] Qiu Z. Shu J. Tang D. (2017). Bioresponsive release system for visual fluorescence detection of carcinoembryonic antigen from mesoporous silica nanocontainers mediated optical color on quantum dot-enzyme-impregnated paper. *Anal. Chem.* 89 5152–5160. 10.1021/acs.analchem.7b00989 28376620

[B160] Qu L. Peng X. (2002). Control of photoluminescence properties of CdSe nanocrystals in growth. *J. Am. Chem. Soc.* 124 2049–2055. 11866620 10.1021/ja017002j

[B161] Qu Y. G. Zhang Q. Pan Q. Zhao X. D. Huang Y. H. Chen F. C. (2014). Quantum dots immunofluorescence histochemical detection of EGFR gene mutations in the non-small cell lung cancers using mutation-specific antibodies. *Int. J. Nanomed.* 9 5771–5778. 10.2147/IJN.S71310 25525358 PMC4266265

[B162] Rabinowits G. Gercel-Taylor C. Day J. M. Taylor D. D. Kloecker G. H. (2009). Exosomal microRNA: a diagnostic marker for lung cancer. *Clin. Lung Cancer* 10 42–46. 10.3816/CLC.2009.n.006 19289371

[B163] Rakovich T. Y. Mahfoud O. K. Mohamed B. M. Prina-Mello A. Crosbie-Staunton K. Van Den Broeck T. (2014). Highly sensitive single domain antibody-quantum dot conjugates for detection of HER2 biomarker in lung and breast cancer cells. *ACS Nano* 8 5682–5695. 10.1021/nn500212h 24873349

[B164] Roush S. Slack F. J. (2008). The let-7 family of microRNAs. *Trends Cell Biol.* 18 505–516. 10.1016/j.tcb.2008.07.007 18774294

[B165] Salzberg T. N. Overstreet B. T. Rogers J. D. Califano J. V. Best A. M. Schenkein H. A. (2006). C-reactive protein levels in patients with aggressive periodontitis. *J. Periodontol.* 77 933–939. 10.1902/jop.2006.050165 16734565

[B166] Saran A. D. Mehra A. Bellare J. R. (2012). Superposition of Quantum Confinement Energy (SQCE) model for estimating shell thickness in core–shell quantum dots: Validation and comparison. *J. Colloid Interface Sci.* 378 21–29. 10.1016/j.jcis.2012.03.056 22578831

[B167] Sarhadi V. K. Wikman H. Salmenkivi K. Kuosma E. Sioris T. Salo J. (2006). Increased expression of high mobility group A proteins in lung cancer. *J. Pathol.* 209 206–212. 10.1002/path.1960 16521118

[B168] Sato F. Tsuchiya S. Terasawa K. Tsujimoto G. (2009). Intra-platform repeatability and inter-platform comparability of microRNA microarray technology. *PLoS One* 4:e5540. 10.1371/journal.pone.0005540 19436744 PMC2677665

[B169] Sauter W. Rosenberger A. Beckmann L. Kropp S. Mittelstrass K. Timofeeva M. (2008). Matrix metalloproteinase 1 (MMP1) is associated with early-onset lung cancer. *Cancer Epidemiol. Biomarkers Prev.* 17 1127–1135. 10.1158/1055-9965.EPI-07-2840 18483334

[B170] Schneider J. (2006). Tumor markers in detection of lung cancer. *Adv. Clin. Chem.* 42 1–41. 17131623 10.1016/s0065-2423(06)42001-1

[B171] Schwarz D. S. Hutvagner G. Du T. Xu Z. Aronin N. Zamore P. D. (2003). Asymmetry in the assembly of the RNAi enzyme complex. *Cell* 115 199–208. 14567917 10.1016/s0092-8674(03)00759-1

[B172] Semina E. Rubina K. Sysoeva V. Rysenkova K. Klimovich P. Plekhanova O. (2016). Urokinase and urokinase receptor participate in regulation of neuronal migration, axon growth and branching. *Eur. J. Cell Biol.* 95 295–310. 10.1016/j.ejcb.2016.05.003 27324124

[B173] Shen X. Xi G. Maile L. A. Wai C. Rosen C. J. Clemmons D. R. (2012). Insulin-like growth factor (IGF) binding protein 2 functions coordinately with receptor protein tyrosine phosphatase beta and the IGF-I receptor to regulate IGF-I-stimulated signaling. *Mol. Cell. Biol.* 32 4116–4130. 10.1128/MCB.01011-12 22869525 PMC3457336

[B174] Shi G. Perle M. A. Mittal K. Chen H. Zou X. Narita M. (2009). Let-7 repression leads to HMGA2 overexpression in uterine leiomyosarcoma. *J. Cell Mol. Med.* 13 3898–3905. 10.1111/j.1582-4934.2008.00541.x 19602040 PMC4516537

[B175] Shibayama T. Ueoka H. Nishii K. Kiura K. Tabata M. Miyatake K. (2001). Complementary roles of pro-gastrin-releasing peptide (ProGRP) and neuron specific enolase (NSE) in diagnosis and prognosis of small-cell lung cancer (SCLC). *Lung Cancer* 32 61–69. 11282430 10.1016/s0169-5002(00)00205-1

[B176] Shu J. Tang D. (2017). Current advances in quantum-dots-based photoelectrochemical immunoassays. *Chem. Asian J.* 12 2780–2789. 10.1002/asia.201701229 28880459

[B177] Sims E. K. Lakhter A. J. Anderson-Baucum E. Kono T. Tong X. Evans-Molina C. (2017). MicroRNA 21 targets BCL2 mRNA to increase apoptosis in rat and human beta cells. *Diabetologia* 60 1057–1065. 10.1007/s00125-017-4237-z 28280903 PMC5425307

[B178] Sithambaram S. Hilmi I. Goh K. L. (2015). The diagnostic accuracy of the M2 pyruvate kinase quick stool test–a rapid office based assay test for the detection of colorectal cancer. *PLoS One* 10:e0131616. 10.1371/journal.pone.0131616 26158845 PMC4497640

[B179] Skog J. Wurdinger T. Van Rijn S. Meijer D. H. Gainche L. Sena-Esteves M. (2008). Glioblastoma microvesicles transport RNA and proteins that promote tumour growth and provide diagnostic biomarkers. *Nat. Cell Biol.* 10 1470–1476. 10.1038/ncb1800 19011622 PMC3423894

[B180] Smith A. M. Nie S. (2009). Next-generation quantum dots. *Nat. Biotechnol.* 27 732–733. 10.1038/nbt0809-732 19668181 PMC2854554

[B181] Sohel M. M. Hoelker M. Noferesti S. S. Salilew-Wondim D. Tholen E. Looft C. (2013). Exosomal and non-exosomal transport of extra-cellular microRNAs in follicular fluid: implications for bovine oocyte developmental competence. *PLoS One* 8:e78505. 10.1371/journal.pone.0078505 24223816 PMC3817212

[B182] Sousa N. G. Cardoso C. R. Silva J. S. Kuga M. C. Tanomaru-Filho M. Faria G. (2014). Association of matrix metalloproteinase inducer (EMMPRIN) with the expression of matrix metalloproteinases-1, -2 and -9 during periapical lesion development. *Arch. Oral. Biol.* 59 944–953. 10.1016/j.archoralbio.2014.05.021 24927330

[B183] Stanisavljevic M. Krizkova S. Vaculovicova M. Kizek R. Adam V. (2015). Quantum dots-fluorescence resonance energy transfer-based nanosensors and their application. *Biosens. Bioelectron.* 74 562–574. 10.1016/j.bios.2015.06.076 26188679

[B184] Stroh M. Zimmer J. P. Duda D. G. Levchenko T. S. Cohen K. S. Brown E. B. (2005). Quantum dots spectrally distinguish multiple species within the tumor milieu in vivo. *Nat. Med.* 11 678–682. 10.1038/nm1247 15880117 PMC2686110

[B185] Su S. Fan J. Xue B. Yuwen L. Liu X. Pan D. (2014). DNA-conjugated quantum dot nanoprobe for high-sensitivity fluorescent detection of DNA and micro-RNA. *ACS Appl. Mater. Interfaces* 6 1152–1157. 10.1021/am404811j 24380365

[B186] Sung H. J. Ahn J. M. Yoon Y. H. Rhim T. Y. Park C. S. Park J. Y. (2011). Identification and validation of SAA as a potential lung cancer biomarker and its involvement in metastatic pathogenesis of lung cancer. *J. Proteome Res.* 10 1383–1395. 10.1021/pr101154j 21141971

[B187] Sung H. J. Jeon S. A. Ahn J. M. Seul K. J. Kim J. Y. Lee J. Y. (2012). Large-scale isotype-specific quantification of Serum amyloid A 1/2 by multiple reaction monitoring in crude sera. *J. Proteomics* 75 2170–2180. 10.1016/j.jprot.2012.01.018 22300576

[B188] Tabet F. Vickers K. C. Cuesta Torres L. F. Wiese C. B. Shoucri B. M. Lambert G. (2014). HDL-transferred microRNA-223 regulates ICAM-1 expression in endothelial cells. *Nat. Commun.* 5:3292. 10.1038/ncomms4292 24576947 PMC4189962

[B189] Takeyama Y. Sato M. Horio M. Hase T. Yoshida K. Yokoyama T. (2010). Knockdown of ZEB1, a master epithelial-to-mesenchymal transition (EMT) gene, suppresses anchorage-independent cell growth of lung cancer cells. *Cancer Lett.* 296 216–224. 10.1016/j.canlet.2010.04.008 20452118 PMC3110825

[B190] Tan A. Yildirimer L. Rajadas J. De La Peña H. Pastorin G. Seifalian A. (2011). Quantum dots and carbon nanotubes in oncology: a review on emerging theranostic applications in nanomedicine. *Nanomedicine* 6 1101–1114. 10.2217/nnm.11.64 21955079

[B191] Tang D. Shen Y. Wang M. Yang R. Wang Z. Sui A. (2013). Identification of plasma microRNAs as novel noninvasive biomarkers for early detection of lung cancer. *Eur. J. Cancer Prev.* 22 540–548. 10.1097/CEJ.0b013e32835f3be9 23462458

[B192] Tang W. Zhu G. Liang L. Zhang C. Y. (2015). A single quantum dot-based biosensor for DNA point mutation assay. *Analyst* 140 5936–5943. 10.1039/c5an01270h 26225372

[B193] Terris B. Cavard C. Perret C. (2010). EpCAM, a new marker for cancer stem cells in hepatocellular carcinoma. *J. Hepatol.* 52 280–281. 10.1016/j.jhep.2009.10.026 20006402

[B194] Thirumalai A. Singh S. K. Hammond D. J. Jr. Gang T. B. Ngwa D. N. Pathak A. (2017). Purification of recombinant C-reactive protein mutants. *J. Immunol. Methods* 443 26–32. 10.1016/j.jim.2017.01.011 28167277

[B195] Tiernan J. P. Perry S. L. Verghese E. T. West N. P. Yeluri S. Jayne D. G. (2013). Carcinoembryonic antigen is the preferred biomarker for in vivo colorectal cancer targeting. *Br. J. Cancer* 108 662–667. 10.1038/bjc.2012.605 23322207 PMC3593555

[B196] Tomita M. Shimizu T. Ayabe T. Yonei A. Onitsuka T. (2010). Prognostic significance of tumour marker index based on preoperative CEA and CYFRA 21-1 in non-small cell lung cancer. *Anticancer Res.* 30 3099–3102. 20683062

[B197] Tsuboi S. Jin T. (2017). Bioluminescence Resonance Energy Transfer (BRET)-coupled annexin v-functionalized quantum dots for near-infrared optical detection of apoptotic cells. *ChemBioChem* 18 2231–2235. 10.1002/cbic.201700486 28901721

[B198] Turchinovich A. Weiz L. Langheinz A. Burwinkel B. (2011). Characterization of extracellular circulating microRNA. *Nucleic Acids Res.* 39 7223–7233. 10.1093/nar/gkr254 21609964 PMC3167594

[B199] Turiak L. Misjak P. Szabo T. G. Aradi B. Paloczi K. Ozohanics O. (2011). Proteomic characterization of thymocyte-derived microvesicles and apoptotic bodies in BALB/c mice. *J. Proteomics* 74 2025–2033. 10.1016/j.jprot.2011.05.023 21635979

[B200] Urieli-Shoval S. Linke R. P. Matzner Y. (2000). Expression and function of serum amyloid A, a major acute-phase protein, in normal and disease states. *Curr. Opin. Hematol.* 7 64–69. 10608507 10.1097/00062752-200001000-00012

[B201] Valadi H. Ekstrom K. Bossios A. Sjostrand M. Lee J. J. Lotvall J. O. (2007). Exosome-mediated transfer of mRNAs and microRNAs is a novel mechanism of genetic exchange between cells. *Nat. Cell Biol.* 9 654–659. 10.1038/ncb1596 17486113

[B202] Valizadeh A. Mikaeili H. Samiei M. Mussa farkhani S. Zarghami N. Kouhi M. (2012). Quantum dots: Synthesis, bioapplications, and toxicity. *Nanoscale Res. Lett.* 7:480. 10.1186/1556-276X-7-480 22929008 PMC3463453

[B203] Vanuytsel T. Vermeire S. Cleynen I. (2013). The role of Haptoglobin and its related protein, Zonulin, in inflammatory bowel disease. *Tissue Barriers* 1:e27321. 10.4161/tisb.27321 24868498 PMC3943850

[B204] Vickers K. C. Palmisano B. T. Shoucri B. M. Shamburek R. D. Remaley A. T. (2011). MicroRNAs are transported in plasma and delivered to recipient cells by high-density lipoproteins. *Nat. Cell Biol.* 13 423–433. 10.1038/ncb2210 21423178 PMC3074610

[B205] Wang B. Xi Y. (2013). Challenges for MicroRNA microarray data analysis. *Microarrays* 2 34–50. 10.3390/microarrays2020034 24163754 PMC3807239

[B206] Wang K. Zhang S. Marzolf B. Troisch P. Brightman A. Hu Z. (2009). Circulating microRNAs, potential biomarkers for drug-induced liver injury. *Proc. Natl. Acad. Sci. U.S.A.* 106 4402–4407. 10.1073/pnas.0813371106 19246379 PMC2657429

[B207] Wang K. Zhang S. Weber J. Baxter D. Galas D. J. (2010). Export of microRNAs and microRNA-protective protein by mammalian cells. *Nucleic Acids Res.* 38 7248–7259. 10.1093/nar/gkq601 20615901 PMC2978372

[B208] Wang L. Li W. Wu B. Li Z. Pan D. Wu M. (2017). Room-temperature synthesis of graphene quantum dots via electron-beam irradiation and their application in cell imaging. *Chem. Eng. J.* 309 374–380.

[B209] Weber J. A. Baxter D. H. Zhang S. Huang D. Y. Huang K. H. Lee M. J. (2010). The microRNA spectrum in 12 body fluids. *Clin. Chem.* 56 1733–1741. 10.1373/clinchem.2010.147405 20847327 PMC4846276

[B210] Wen K. C. Sung P. L. Chou Y. T. Pan C. M. Wang P. H. Lee O. K. (2018). The role of EpCAM in tumor progression and the clinical prognosis of endometrial carcinoma. *Gynecol. Oncol.* 148 383–392. 10.1016/j.ygyno.2017.11.033 29208367

[B211] Weng Y. Li Z. Peng L. Zhang W. Chen G. (2017). Fabricating carbon quantum dots with nano-defined position and pattern in one step via sugar-electron-beam-writing. *Nanoscale* 9 19263–19270. 10.1039/c7nr07892g 29188850

[B212] Widschwendter M. Evans I. Jones A. Ghazali S. Reisel D. Ryan A. (2017). Methylation patterns in serum DNA for early identification of disseminated breast cancer. *Genome Med.* 9:115. 10.1186/s13073-017-0499-9 29268762 PMC5740791

[B213] Wildstein K. A. Faustini Y. Yip R. Henschke C. I. Ostroff J. S. (2011). Longitudinal predictors of adherence to annual follow-up in a lung cancer screening programme. *J. Med. Screen* 18 154–159. 10.1258/jms.2011.010127 22045825

[B214] Wong M. C. S. Lao X. Q. Ho K. F. Goggins W. B. Tse S. L. A. (2017). Incidence and mortality of lung cancer: global trends and association with socioeconomic status. *Sci. Rep.* 7:14300. 10.1038/s41598-017-14513-7 29085026 PMC5662733

[B215] Wong T. S. Man O. Y. Tsang C. M. Tsao S. W. Tsang R. K. Chan J. Y. (2011). MicroRNA let-7 suppresses nasopharyngeal carcinoma cells proliferation through downregulating c-Myc expression. *J. Cancer Res. Clin. Oncol.* 137 415–422. 10.1007/s00432-010-0898-4 20440510 PMC3036828

[B216] Wu C. Liu J. Zhang P. Li J. Ji H. Yang X. (2015). A recognition-before-labeling strategy for sensitive detection of lung cancer cells with a quantumdot-aptamer complex. *Analyst* 140 6100–6107. 10.1039/c5an01145k 26200911

[B217] Wu D. Hu Y. Tong S. Williams B. R. Smyth G. K. Gantier M. P. (2013). The use of miRNA microarrays for the analysis of cancer samples with global miRNA decrease. *RNA* 19 876–888. 10.1261/rna.035055.112 23709276 PMC3683922

[B218] Wu S. Liu L. Li G. Jing F. Mao H. Jin Q. (2016). Multiplexed detection of lung cancer biomarkers based on quantum dots and microbeads. *Talanta* 15 48–54. 10.1016/j.talanta.2016.05.005 27260434

[B219] Xia X. Lu J. J. Zhang S. S. Su C. H. Luo H. H. (2016). Midkine is a serum and urinary biomarker for the detection and prognosis of non-small cell lung cancer. *Oncotarget* 7 87462–87472. 10.18632/oncotarget.13865 27974680 PMC5350001

[B220] Xiao K. Fang Z. Gao X. Zhao J. Huang R. Xie M. (2017). Membrane complement regulatory protein reduces the damage of transplanting autologous bone marrow mesenchymal stem cells by suppressing the activation of complement. *Injury* 48 2089–2094. 10.1016/j.injury.2017.08.008 28823400

[B221] Xin H. Li Y. Buller B. Katakowski M. Zhang Y. Wang X. (2012). Exosome-mediated transfer of miR-133b from multipotent mesenchymal stromal cells to neural cells contributes to neurite outgrowth. *Stem Cells* 30 1556–1564. 10.1002/stem.1129 22605481 PMC3495063

[B222] Xing L. Todd N. W. Yu L. Fang H. Jiang F. (2010). Early detection of squamous cell lung cancer in sputum by a panel of microRNA markers. *Mod. Pathol.* 23 1157–1164. 10.1038/modpathol.2010.111 20526284

[B223] Yanez-Mo M. Siljander P. R. Andreu Z. Zavec A. B. Borras F. E. Buzas E. I. (2015). Biological properties of extracellular vesicles and their physiological functions. *J. Extracell. Vesicles* 4:27066. 10.3402/jev.v4.27066 25979354 PMC4433489

[B224] Yang P. Matras-Postołek K. Song X. Zheng Y. Liu Y. Ding K. (2015). Self-assembly and photoluminescence evolution of hydrophilic and hydrophobic quantum dots in sol-gel processes. *Mater. Res. Bull.* 70 385–391. 10.1016/j.materresbull.2015.04.051

[B225] Yang Y. Meng H. Peng Q. Yang X. Gan R. Zhao L. (2015). Downregulation of microRNA-21 expression restrains non-small cell lung cancer cell proliferation and migration through upregulation of programmed cell death 4. *Cancer Gene Ther.* 22 23–29. 10.1038/cgt.2014.66 25477028

[B226] Yoshida Y. Ikematsu S. Moritoyo T. Goto M. Tsutsui J. Sakuma S. (2001). Intraventricular administration of the neurotrophic factor midkine ameliorates hippocampal delayed neuronal death following transient forebrain ischemia in gerbils. *Brain Res.* 894 46–55. 11245814 10.1016/s0006-8993(00)03209-1

[B227] Yousif A. M. Ingangi V. Merlino F. Brancaccio D. Minopoli M. Bellavita R. (2018). Urokinase receptor derived peptides as potent inhibitors of the formyl peptide receptor type 1-triggered cell migration. *Eur. J. Med. Chem.* 143 348–360. 10.1016/j.ejmech.2017.11.030 29202399

[B228] Yuxia M. Zhennan T. Wei Z. (2012). Circulating miR-125b is a novel biomarker for screening non-small-cell lung cancer and predicts poor prognosis. *J. Cancer Res. Clin. Oncol.* 138 2045–2050. 10.1007/s00432-012-1285-0 22806310 PMC11824208

[B229] Zeng Y. P. Zhu G. Yang X. Y. Cao J. Jing Z. L. Zhang C. Y. (2014). A quantum dot-based microRNA nanosensor for point mutation assays. *Chem. Commun.* 50 7160–7162. 10.1039/c4cc02034k 24853117

[B230] Zernecke A. Bidzhekov K. Noels H. Shagdarsuren E. Gan L. Denecke B. (2009). Delivery of microRNA-126 by apoptotic bodies induces CXCL12-dependent vascular protection. *Sci. Signal.* 2:ra81. 10.1126/scisignal.2000610 19996457

[B231] Zhang B. Pan X. Cobb G. P. Anderson T. A. (2007). microRNAs as oncogenes and tumor suppressors. *Dev. Biol.* 302 1–12. 10.1016/j.ydbio.2006.08.028 16989803

[B232] Zhang B. Shen L. Shi H. Pan Z. Wu L. Yan Y. (2016). Exosomes from human umbilical cord mesenchymal stem cells: identification, purification, and biological characteristics. *Stem Cells Int.* 2016:1929536. 10.1155/2016/1929536 28105054 PMC5220513

[B233] Zhang C. Ji X. Zhang Y. Zhou G. Ke X. Wang H. (2013). One-pot synthesized aptamer-functionalized CdTe:Zn2+ quantum dots for tumor-targeted fluorescence imaging in vitro and in vivo. *Anal. Chem.* 85 5843–5849. 10.1021/ac400606e 23682757

[B234] Zhang Y. Ying X. Han S. Wang J. Zhou X. Bai E. (2013). Autoantibodies against insulin-like growth factor binding protein-2 as a serological biomarker in the diagnosis of lung cancer. *Int. J. Oncol.* 42 93–100. 10.3892/ijo.2012.1699 23165420 PMC3583617

[B235] Zhang H. Wang Y. Zhao D. Zeng D. Xia J. Aldalbahi A. (2015). Universal fluorescence biosensor platform based on graphene quantum dots and pyrene-functionalized molecular beacons for detection of MicroRNAs. *ACS Appl. Mater. Interfaces* 7 16152–16156. 10.1021/acsami.5b04773 26200323

[B236] Zhang J. G. Wang J. J. Zhao F. Liu Q. Jiang K. Yang G. H. (2010). MicroRNA-21 (miR-21) represses tumor suppressor PTEN and promotes growth and invasion in non-small cell lung cancer (NSCLC). *Clin. Chim. Acta* 411 846–852. 10.1016/j.cca.2010.02.074 20223231

[B237] Zhang Y. Liu D. Chen X. Li J. Li L. Bian Z. (2010). Secreted monocytic miR-150 enhances targeted endothelial cell migration. *Mol. Cell.* 39 133–144. 10.1016/j.molcel.2010.06.010 20603081

[B238] Zhang K. Lv S. Lin Z. Tang D. (2017). CdS:Mn quantum dot-functionalized g-C3N4 nanohybrids as signal-generation tags for photoelectrochemical immunoassay of prostate specific antigen coupling DNAzymeconcatamer with enzymatic biocatalytic precipitation. *Biosens. Bioelectron.* 95 34–40. 10.1016/j.bios.2017.04.005 28412658

[B239] Zhao H. Shen J. Medico L. Wang D. Ambrosone C. B. Liu S. (2010). A pilot study of circulating miRNAs as potential biomarkers of early stage breast cancer. *PLoS One* 5:e13735. 10.1371/journal.pone.0013735 21060830 PMC2966402

[B240] Zhao N. Liu S. Jiang Q. Lan T. Cheng Z. Liu H. (2016). Small-Protein-stabilized semiconductor nanoprobe for targeted imaging of cancer cells. *Chembiochem* 17 1202–1206. 10.1002/cbic.201600219 27123671

[B241] Zhao Y. Chen F. Li Q. Wang L. Fan C. (2015). Isothermal amplification of nucleic acids. *Chem. Rev.* 115 12491–12545. 10.1021/acs.chemrev.5b00428 26551336

[B242] Zhao Y. Ruan X. Wang H. Li X. Gu M. Wang L. (2017). The presence of a membrane-bound progesterone receptor induces growth of breast cancer with norethisterone but not with progesterone: a xenograft model. *Maturitas* 102 26–33. 10.1016/j.maturitas.2017.05.007 28610679

